# Molecular interactions on single-walled carbon nanotubes revealed by high-resolution transmission microscopy

**DOI:** 10.1038/ncomms8732

**Published:** 2015-07-15

**Authors:** Tomokazu Umeyama, Jinseok Baek, Yuta Sato, Kazu Suenaga, Fawzi Abou-Chahine, Nikolai V. Tkachenko, Helge Lemmetyinen, Hiroshi Imahori

**Affiliations:** 1 Department of Molecular Engineering, Graduate School of Engineering, Kyoto University, Nishikyo-ku, Kyoto 615-8510, Japan.; 2 Nanomaterials Research Institute, National Institute of Advanced Industrial Science and Technology (AIST), AIST Central 5, Tsukuba 305-8565, Japan.; 3 Department of Chemistry and Bioengineering, Tampere University of Technology, P.O. Box 541, FIN-33101 Tampere, Finland.; 4 Institute for Integrated Cell-Material Sciences (WPI-iCeMS), Kyoto University, Nishikyo-ku, Kyoto 615-8510, Japan.

## Abstract

The close solid-state structure–property relationships of organic π−aromatic molecules have attracted interest due to their implications for the design of organic functional materials. In particular, a dimeric structure, that is, a unit consisting of two molecules, is required for precisely evaluating intermolecular interactions. Here, we show that the sidewall of a single-walled carbon nanotube (SWNT) represents a unique molecular dimer platform that can be directly visualized using high-resolution transmission electron microscopy. Pyrene is chosen as the π−aromatic molecule; its dimer is covalently linked to the SWNT sidewalls by aryl addition. Reflecting the orientation and separation of the two molecules, the pyrene dimer on the SWNT exhibits characteristic optical and photophysical properties. The methodology discussed here—form and probe molecular dimers—is highly promising for the creation of unique models and provides indispensable and fundamental information regarding molecular interactions.

## Abstract (web summary)

Probing local molecular properties is crucial for the rational designs of functional organic materials. Here, Umeyama *et al*. prepare a dimeric structure of a model π-aromatic compound on the sidewall of a carbon nanotube to be visualized by transmission electron microscopy at a single-molecule level.

## Graphical abstract

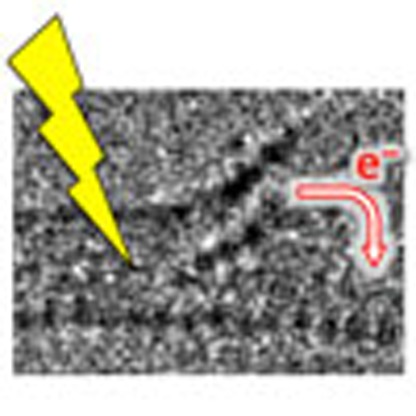

Probing local molecular structures and properties can provide information that is extremely valuable for material scientists. X-ray diffraction is a powerful technique for determining precise molecular structures and packing at the atomic level. This technique, however, requires the use of large-size single crystals, which are often difficult to grow. Moreover, the regular molecular arrangements found in crystals differ from the irregular arrangements that are seen in amorphous states. Therefore, an alternative methodology to determine local molecular structures and properties is highly desirable. Recent remarkable progress using high-resolution transmission electron microscopy (HR-TEM) technology has enabled the visualization of the structures^1^,^2^,^3^, movements^4^,^5^,^6^ and reactions^7^,^8^,^9^,^10^ of single small organic molecules. However, the visualization of precise molecular amorphous structures at the atomic level remains challenging. The electronic structures and physical properties of organic π-aromatic materials can be altered by molecular interactions in the solid-state as well as intrinsic structures and properties as monomers. Elucidation of the close relationship between the solid-state local structure and the physical properties of π−aromatic molecules is of great scientific and technological interest^11^,^12^.

Herein we focus on the sidewall of a single-walled carbon nanotube (SWNT)^13^,^14^,^15^ and its use as a unique platform to examine ‘the smallest molecular assembly' (dimers), which can then be fully characterized microscopically, spectroscopically and theoretically. Theoretical calculations predicted that reactions with aryl radicals will lead to association of the attached aryl positions on the SWNT sidewalls^16^, although no experimental evidence is available regarding the binding mode. According to the calculations, then proximity is due to the binding of a single aryl group with an unpaired electron that activates carbon atoms near the binding site, thereby accelerating the addition of the next aryl group at the nearby carbon atoms^17^. On the basis of this interaction model, we successfully synthesize the dimeric structure of a model π-aromatic compound, pyrene^18^, on SWNTs; the dimer is then visualized for the first time at the single-molecule level using HR-TEM measurements and is probed spectroscopically to evaluate the close structure–property relationships.

## Results

### Formation of monomeric and dimeric pyrenes on SWNT

**Figure 1:**
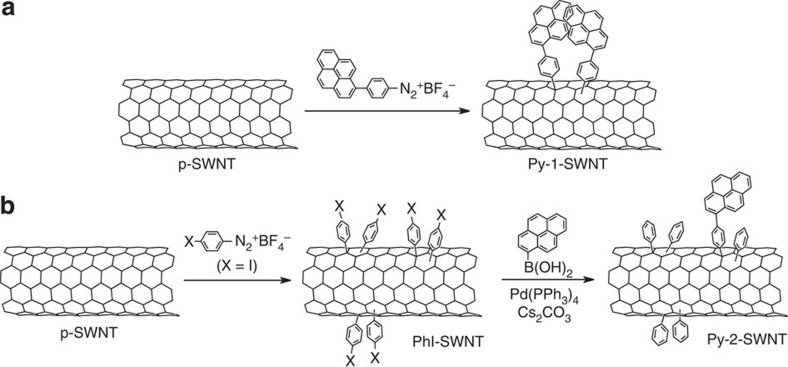
Schematic representation of the functionalization of SWNTs with pyrene. (**a**) One-step functionalization of p-SWNT. The reaction of p-SWNT with PP radicals generated from the diazonium salt yielded Py-1-SWNT. Two PP groups are expected to exist in proximity to one another. (**b**) Two-step functionalization of p-SWNT. PhI-SWNT was prepared by treating of p-SWNT with *p*-iodobenzenediazonium salt. Then, the Suzuki coupling reaction of PhI-SWNT with pyrenyl boronic ester yielded Py-2-SWNT. The low yield (9%) of the Suzuki coupling reaction ensures that the pyrenyl groups are monomeric.

As shown in Fig. 1a, pyrenes were attached to purified SWNTs (p-SWNT) using a one-step method, that is, the direct addition of 4-(1-pyrenyl)phenyl (PP) radicals onto the SWNT sidewall (Py-1-SWNT). Pairwise addition of PP groups onto the SWNTs is expected based on theoretical predictions^16^. To estimate the functionalization ratio of Py-1-SWNT, a thermogravimetric analysis (TGA) was conducted under a nitrogen atmosphere (Supplementary Fig. 1). The TGA trace of Py-1-SWNT reveals a two-step weight loss, which reflects detachment of the covalently linked PP groups from the sidewalls before SWNT combustion. A turning point, where the slope becomes the minimum between the two weight loss events, is at ∼450 °C. The relative weight loss of Py-1-SWNT was 7.2% at the turning point, corresponding to one PP moiety per ∼240 carbon atoms of the SWNT. This estimation predicts the existence of one plausible pair of PP groups per ∼2.0 nm of nanotube length. Considering the size of the PP unit (1.14 nm, Supplementary Fig. 2) and the curved surface structure of the SWNTs, pyrene pairs on the SWNT are likely isolated from one another. However, the TGA trace around the turning point is not horizontal but gently downward-sloping, indicating incomplete detachment of the PP groups at approximately 450 °C. Thus, the functionalization ratio of Py-1-SWNT might be underestimated using the TGA method.

For comparison, we also synthesized monomeric pyrenes on SWNT sidewalls selectively using a two-step method, that is, a Suzuki coupling reaction between a pre-prepared iodophenyl-functionalized SWNT (PhI-SWNT) and a pyrene boronic ester (Py-2-SWNT, Fig. 1b). X-ray photoelectron spectroscopy (XPS) measurements of PhI-SWNT revealed signals attributable to iodine (2.1%), thereby enabling the estimation of the functionalization ratio of PhI-SWNT (Supplementary Fig. 3); the resulting value corresponded to one PhI group per ∼42 carbon atoms of the SWNT. This value is considerably larger than that estimated using TGA (one PhI moiety per ∼52 carbons of SWNT, Supplementary Fig. 1). The existence of the residual PhI signal at the turning point (450 °C) might have caused the functionalization ratio to have been underestimated by the TGA method. The Suzuki coupling reaction of the PhI-SWNT with pyrenyl boronic ester yielded pyrene-tethered SWNT (Py-2-SWNT). After this reaction, the signals attributed to iodine were completely lost in the XPS measurements (Supplementary Fig. 3). The TGA measurements, however, revealed a functionalization ratio of one PP group per ∼450 carbon atoms of the SWNT (Supplementary Fig. 1). Thus, the reaction yield of the Suzuki coupling on the SWNT sidewall was 9%. The remaining iodine atoms in the PhI groups were consumed by an incomplete Suzuki coupling reaction. The low yield (9%) of the Suzuki coupling reaction would render the PP groups monomeric in Py-2-SWNT, even if the PhI groups in the PhI-SWNT formed paired structures on the SWNT.

All of the functionalized SWNTs (Py-1-SWNT, PhI-SWNT and Py-2-SWNT) exhibited sufficient dispersibility in common organic solvents, including *N,N*-dimethylformamide (DMF), for spectroscopic measurement. The resulting dark brown solutions exhibited no discernible particulate matter and remained stable for at least a few months. Moreover, the structures of these functionalized SWNTs were further evaluated using atomic force microscopy (AFM, Supplementary Fig. 4) and resonant Raman spectroscopy (Supplementary Fig. 5).

**Figure 2:**
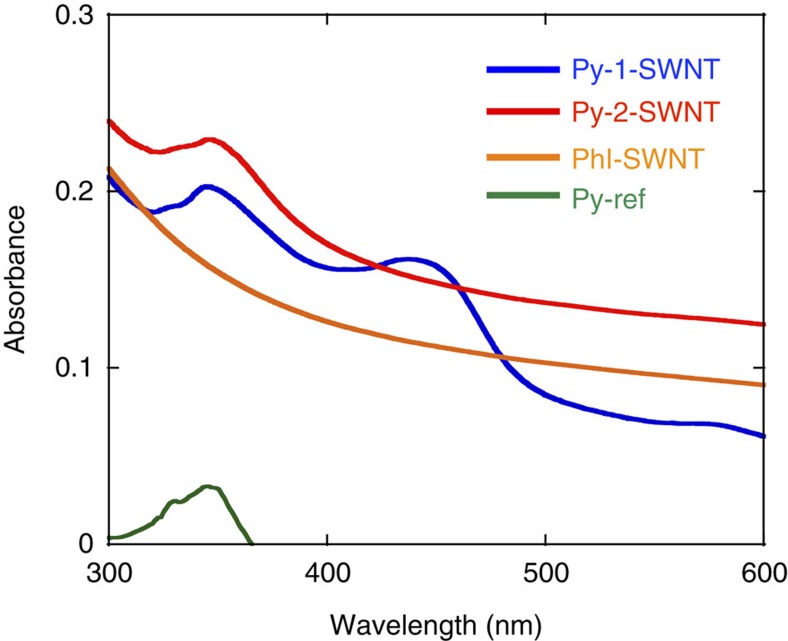
UV-visible absorption spectra. Spectra of Py-1-SWNT (blue line), Py-2-SWNT (red line), PhI-SWNT (orange line) and Py-ref (0.71 μM, green line) were recorded in DMF. The distinct additional band at 450 nm observed for Py-1-SWNT verifies the pairwise addition of PP onto the SWNT.

The dimeric and monomeric nature of the structures located on the SWNT sidewalls in Py-1-SWNT and Py-2-SWNT, respectively, were substantiated using UV–vis absorption measurements. Figure 2 shows the absorption spectra of Py-1-SWNT, Py-2-SWNT, PhI-SWNT and Py-ref (1-phenylpyrene) in DMF. The spectrum of Py-2-SWNT contains features corresponding to both the PhI-SWNT and Py-ref spectra; that is, a pyrene π−π* band at 350 nm and a broad structureless absorption band extending to the near-infrared (NIR) region corresponding to the SWNT. This result was consistent with the presence of monomeric pyrenes in Py-2-SWNT. In contrast, the Py-1-SWNT spectrum exhibited a band at 450 nm with an intensity comparable to that of the π−π* band at 350 nm. This new peak suggests the existence of a dimeric interaction between the pyrene rings in Py-1-SWNT. Typically two split energy levels are present in the excited states of the dimers of conjugated compounds, and the strength of the electronic transition that depends on the transition dipole moment to the two states of the electronically coupled molecules is the vector sum of the transition dipole moments of the individual molecules^19^. Therefore, the existence of two absorption bands in the spectrum of Py-1-SWNT can be rationalized by the formation of pyrene dimers in Py-1-SWNT. A similar absorption split has been previously observed in a pyrene dimer model compound, that is, pyrenophane^18^,^20^,^21^, which exhibits a rotation angle of ∼60°.

### Precise structural characterizations of pyrenes on SWNT

**Figure 3:**
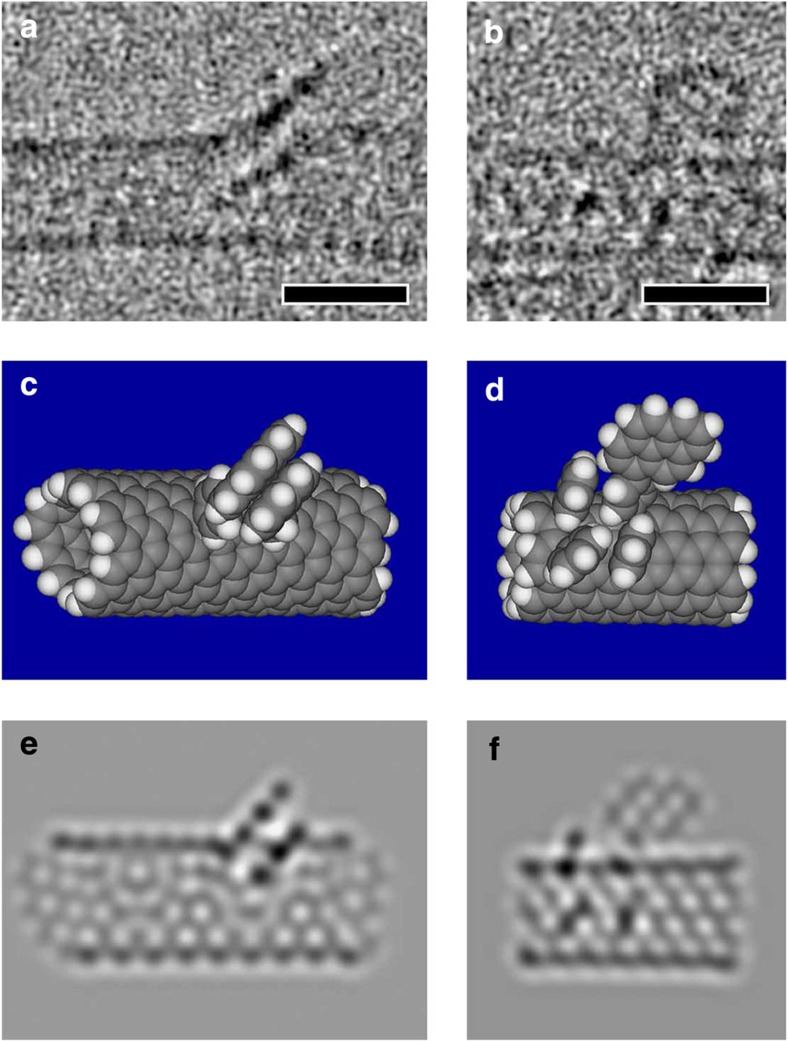
Precise structural characterization. (**a**,**b**) HR-TEM images of (**a**) Py-1-SWNT and (**b**) Py-2-SWNT. Scale bar,1 nm. (**c**,**d**) Model structures of (**c**) paired PP groups on the (9,4)SWNT model and (**d**) one PP and three phenyl groups on the (6,6)SWNT model optimized by DFT calculations at the RB97D/3-21G* level. (**e**,**f**) Corresponding image simulations of HR-TEM are shown in (**e**) and (**f**).

HR-TEM measurements at a reduced electron-beam energy of 60 kV complemented the previously described methods in describing monomeric and dimeric structures located on the SWNT sidewalls in Py-1-SWNT and Py-2-SWNT. The dimeric structure of the pyrenes as they stacked on one another was unambiguously visualized for Py-1-SWNT (Fig. 3a). The distance between the pyrenyl rings is ∼3.4 Å, a value that is comparable to that found between graphene planes in graphite. The stability of the pyrene stacking was corroborated by analysing sequential HR-TEM images; the pyrenes did not separate even though the SWNT scaffold vibrates due to the electron-beam energy used (Supplementary Fig. 6). In addition, sequential HR-TEM images of Py-2-SWNT revealed that the monomeric pyrene was not tilted but was upright on the SWNT sidewall due to presence of the rigid, short phenylene spacer (Supplementary Fig. 7). This is the first example of the visualization of individual planar polycyclic aromatic compounds linked covalently to the exterior surface of nanocarbon scaffolds are individually visualized^2^,^22^,^23^.

**Figure 4:**
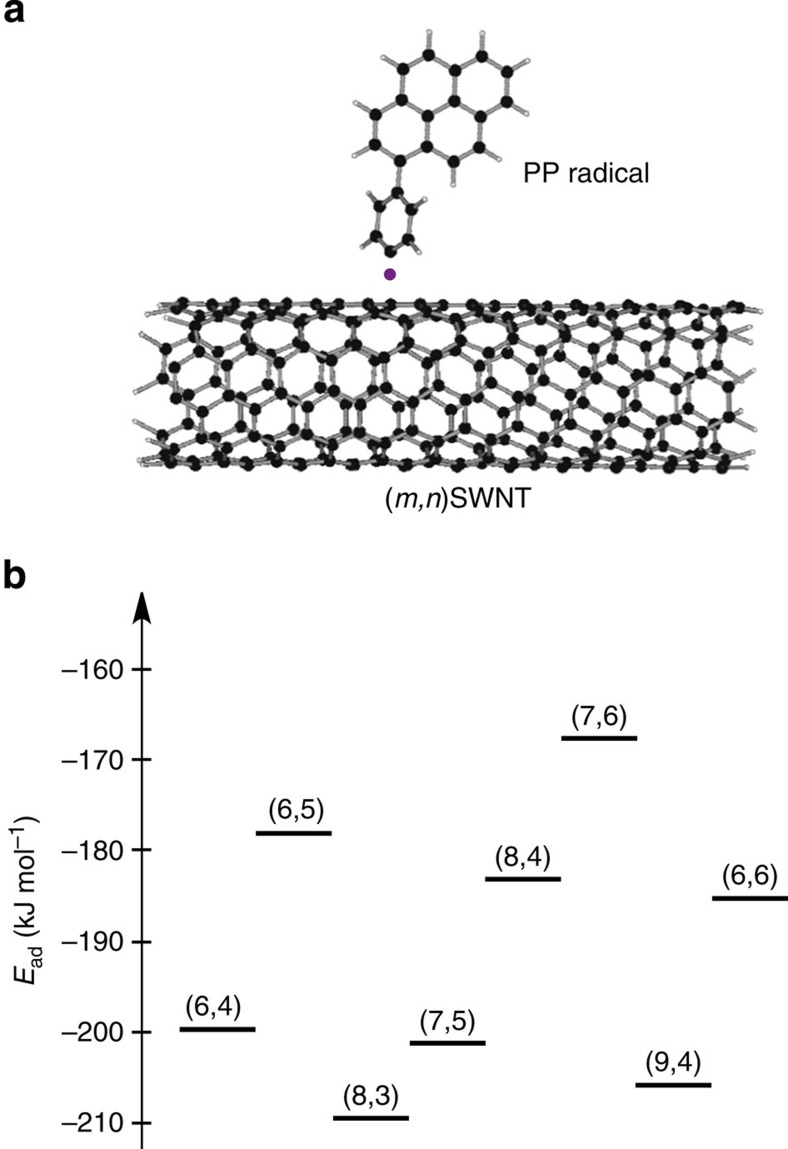
The primary addition of one PP radical onto a SWNT model. (**a**) Schematic image. The purple dot denotes an unpaired electron. (**b**) Energies of the different configurations possible for the primary attachment of a single PP radical on the SWNT models with various chiral indices estimated via the DFT calculations at the RB97D/3-21G* level. The calculated chiral indices were selected based on the chiral composition estimated based on the absorption spectrum shown in Supplementary Fig. 8. The calculation was also conducted using a (10,2)SWNT model but did not converge. The binding energy of the first PP radical was determined according to *E*_ad_=*E*(PP/(m,n)SWNT)–*E*((m,n)SWNT)–*E*(PP). The condensed SWNTs in Py-1-SWNT relative to p-SWNT [(8,3), (7,5), and (9,4)SWNTs (Supplementary Figs 8 and 9)] exhibit relatively large |*E*_ad_| values. The aryl addition and purification processes enriched these SWNTs because less reactive SWNTs were less dispersible in the organic solvents and were therefore removed during centrifugation. However, although the percentage of metallic SWNTs in the p-SWNT is low (<5%) and the |*E*_ad_| for (6,6)SWNT is not large, the kinetically high reactivity^13^ might enrich the metallic SWNTs after the reaction and purification processes.

HR-TEM images recorded at atomic resolution enabled us to illustrate the precise structure of the pyrene dimers attached to the SWNT sidewall in Py-1-SWNT. We determined that the chiral index of the SWNT in Fig. 3a is (9,4) based on the diameters and fringes. The chiral compositions estimated using the peak area ratios in the absorption spectra of p-SWNT (Supplementary Fig. 8) and Py-1-SWNT (Supplementary Fig. 9) and the relative energy of the first attachment of the PP radical estimated using the DFT calculations (Fig. 4) rationalize the counterintuitive appearance of (9,4)SWNT as typical under the harsh HR-TEM conditions. Next, based on the dimeric structure shown in Fig. 3a, we constructed a model of the pyrene dimer on a unit cell of (9,4)SWNT that was terminated with hydrogen atoms as depicted in Fig. 3c; the two PP groups are tethered at the carbons of (9,4)SWNT at a distance of five C-C bonds towards the axial direction. Note here that the geometry was optimized using DFT calculations at RB97D/3-21G* level. On the basis of this model, image simulation was performed for HR-TEM (Fig. 3e). The shape and the contrasting density of the experimental image shown in Fig. 3a are well reproduced in this simulated image.

**Figure 5:**
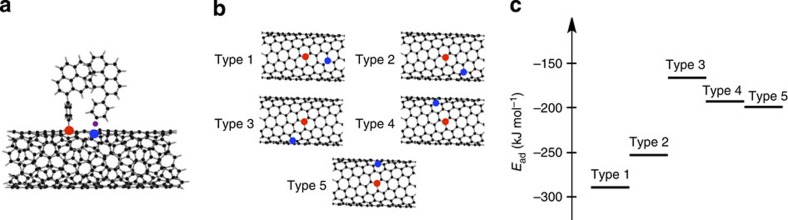
The second addition of a PP radical to the (9,4)SWNT model. (**a**) Schematic image. (**b**) Different configurations of the second attachment of the PP radical on the (9,4)SWNT model (C_520_H_20_). Red and blue circles represent the sites for the first and second attachments, respectively (the purple dot in **a** denotes an unpaired electron). (**c**) Energies of alternative configurations for the second attachment of the PP radical on the (9,4)SWNT model as estimated using DFT calculations at the RB97D/3-21G* level. The binding energy of the second PP was determined according to *E*_ad_=*E*(2PP/(9,4)SWNT)−*E*(PP/(9,4)SWNT)−*E*(PP). Note here that the type 1 configuration with the lowest *E*_ad_ corresponds to the model structure shown in Fig. 3c.

In sharp contrast to small-molecule scaffolds such as naphthalene, SWNTs offer many tethering points^24^,^25^. This renders it difficult to unambiguously identify the precise structure of the pyrene dimer solely from the HR-TEM images. Thus, we used the information obtained from theoretical calculations to exclude other possible structures. Considering the bulky nature of the PP groups, the sites that are four or five bonds away from the first PP attachment site are the most plausible for the second PP attachment. To assess the binding energies of the PP dimer to (9,4)SWNT, we conducted DFT calculations at the RB97D/3-21G* level using the unit cell model of (9,4)SWNT. Reaction at carbon atoms four bonds distant in (9,4)SWNT yielded a less stable configuration, consistent with analogous calculations for graphene sheets^17^. Functionalization at carbon atoms five C-C bonds distant in the axial direction afforded the most stable configuration of the dimeric PP-functionalized (9,4)SWNT based on thermodynamic considerations (type 1 in Fig. 5). The ineffective π−π stacking between the pyrene units might render circumferential addition less stable than axial attachment. These theoretical arguments agree well with the model presented in Fig. 3c.

Typically, the absorption spectra of *J*-aggregates of planar conjugated molecules are bathochromically shifted compared with the corresponding monomeric spectra^19^. Thus, the presence of the two comparable bands in the absorption spectrum of Py-1-SWNT (Fig. 2) might also be rationalized by the existence of pyrene monomers and dimers with a slipped stacked *J*-type configuration in Py-1-SWNT. However, this possibility can be denied because stable model structures of pyrene dimers with *J*-type interactions cannot be constructed on SWNTs.

For the reference Py-2-SWNT system (Fig. 3b), the pyrenyl units were introduced via the Suzuki coupling reaction between PhI-SWNT and pyrenyl boronic ester. Thus, the positional relationship between the PhI groups in the PhI-SWNT is of great significance for the precise characterization of Py-2-SWNT. Unfortunately, we could not detect the PhI groups in the PhI-SWNT sample using HR-TEM due to their small size relative to the pyrenes. To assess the binding energies of the PhI pairs in various configurations, we performed DFT calculations using a finite (6,6)SWNT model terminated with hydrogen atoms (C_168_H_24_) (Supplementary Fig. 10). The chiral index of the SWNT shown in Fig. 3b is (6,6) (Fig. 4 and Supplementary Figs 8 and 9). Again, sites at an even-number (2 and 4) of bonds away from the first attachment site were found unstable. The energetically favoured configurations (type 7–9 in Supplementary Fig. 10) possess the two PhI groups at *para* and *ortho* positions in the same hexagon, and the difference in the direction of the reacted carbons (axial or circumferential) has little influence on the binding energies. Next, we conducted DFT calculations to estimate the optimized geometry of the (6,6)SWNT model including one PP–phenyl pair and one phenyl–phenyl pair at *para* positions with circumferential directions; the result is shown in Fig. 3d. The experimental HR-TEM image of Py-2-SWNT shown in Fig. 3b is well reproduced by the simulated image of model (Fig. 3f). However, there are no further experimental or theoretical indications for the exclusive formation of the circumferential *para* configuration; thus, other structures, such as the *para* configuration in the axial direction and *ortho* configurations, might exist for Py-2-SWNT.

### Relation between dimeric structures and optical properties

**Figure 6:**
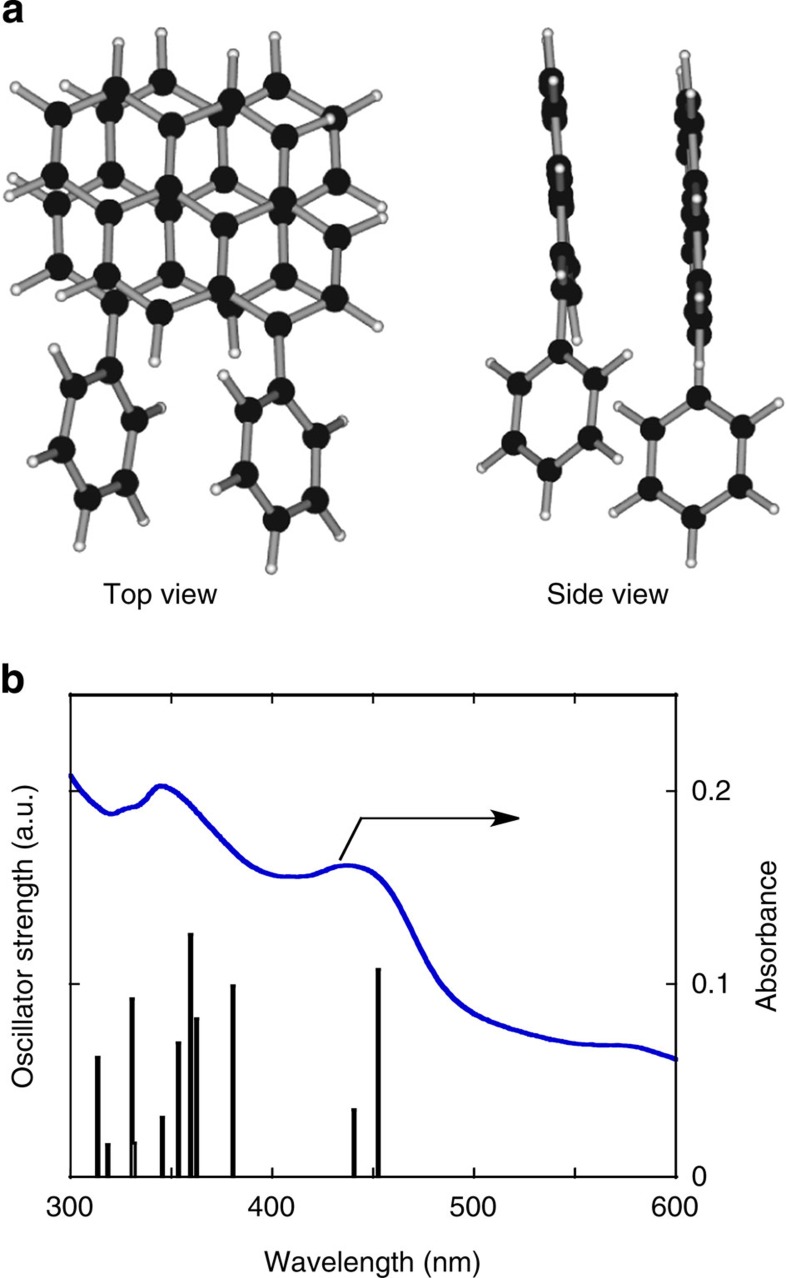
Structure and oscillation intensity of the 1-phenylpyrene pair. (**a**) Structure of the PP pair contained in the model shown in Fig. 3c. The intersecting angle between the two pyrene planes is 67°. (**b**) Oscillator strength versus electronic transition energies for the PP pair. The experimental UV-visible absorption spectrum of Py-1-SWNT, as depicted in Fig. 2, is shown for comparison.

To evaluate correlations between the pyrene dimeric structure and its absorption properties, the optimized dimeric structure (Fig. 6a) taken from the Py-1-SWNT model of type 1 configuration was used to calculate the electronic transition energy via TD-DFT at the RB3LYP/6-31G level. The calculation result and the experimental absorption spectrum of Py-1-SWNT are shown in Fig. 6b and agree well; the simulation predicts a peak at ∼450 nm with an oscillator strength comparable to that of the peak at 360 nm. In contrast, Py-1-SWNT models with different configurations of type 2–5 exhibit only weak or no significant oscillator strength at approximately 450 nm (Supplementary Fig. 11). These models are less satisfactory at reproducing the experimental absorption profiles.

The frontier orbital contour plots for the PP dimers separated from the Py-1-SWNT models with configurations of type 1–3 are depicted together with Py-ref in Supplementary Figs 12–15. These plots suggest the existence of extensive π–π interactions between the pyrenes in the type 1 (Supplementary Fig. 12) and type 2 (Supplementary Fig. 13) dimers. However, the orbital coefficients of the type 3 pyrene dimer are localized on one pyrene (Supplementary Fig. 14), similar to the result found for Py-ref (Supplementary Fig. 15). This result suggests that no pyrene–pyrene interaction occurs in the dimer of type 3 configuration. Note that the distances between the pyrenes in the dimers of the type 4 and 5 configuration are larger than that in the dimer of type 3 configuration, yielding no pyrene–pyrene interactions in the type 4 and 5 models. The excitation energies, oscillator strengths and compositions of the dimer models with configurations of type 1 (Supplementary Fig. 16) and type 2 (Supplementary Fig. 17) were obtained using TD-DFT calculations. In both these cases, the transition with the lowest energy is primarily derived from the HOMO to LUMO transition. It is apparent that the newly emerging absorption band at 450 nm that is observed in the experimental absorption spectrum of Py-1-SWNT agrees better with the type 1 model than the type 2 model. The observed differences in the absorption spectroscopic features might result from differences in the degree of through-space interactions between the pyrenes in the type 1 and type 2 models because the pyrene–pyrene stacking angle is 67° for type 1 and 50° for type 2 (ref. 26).

Importantly, the UV-visible absorption spectrum reflects a statistically averaged structure of the dimeric pyrenes in a bulk sample of Py-1-SWNT, whereas the TEM measurements reveal a localized, individual pyrene-stacking structure. The good accordance between the experimental and calculated UV-visible absorption spectra shown in Fig. 6b implies that the dimeric structure observed via HR-TEM in Fig. 3a represents the real distribution of the structure for the entire Py-1-SWNT sample. The relatively high attraction interaction to form the pyrene stacking structure with a rotational angle of 67° during the second addition of the PP radical to SWNT might contribute to the exclusive selectivity of the configuration. Furthermore, the clear correspondence between the HR-TEM image and the UV-visible absorption result parallels agreement between the structures of the pyrene dimers obtained under the high-vacuum HR-TEM condition and that obtained in the solution state.

### Photophysics

**Figure 7:**
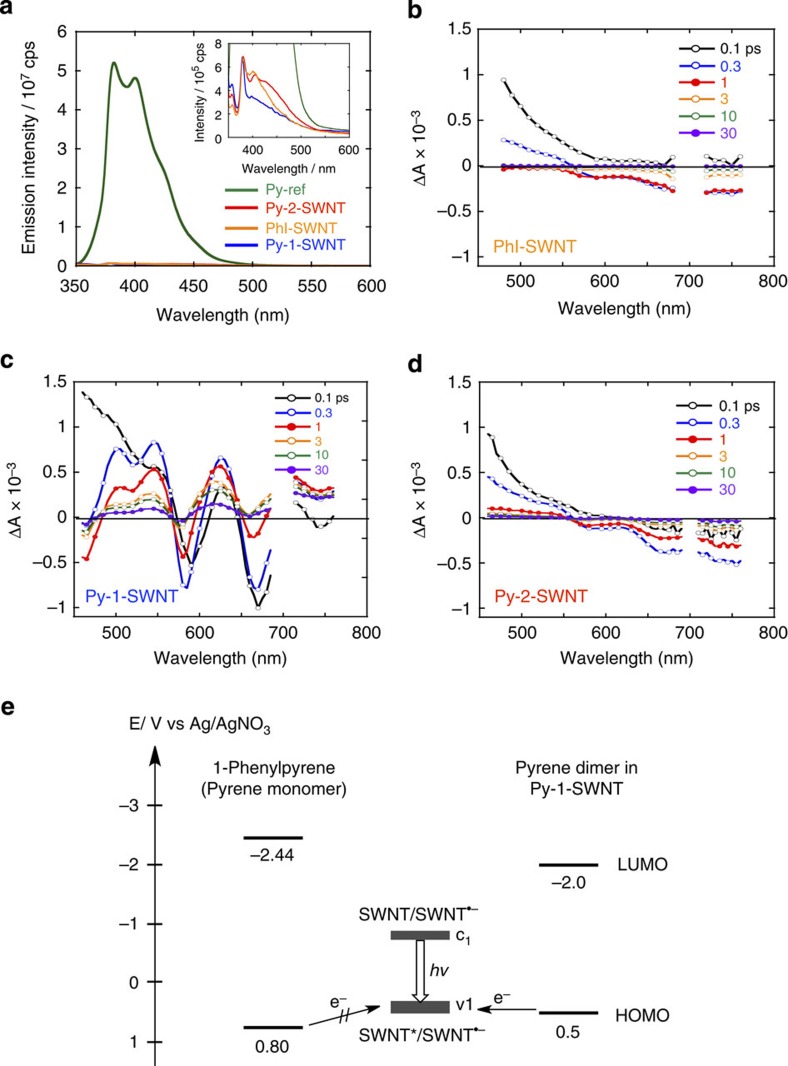
Photodynamics study. (**a**) Fluorescence spectra of Py-1-SWNT (blue line), PhI-SWNT (orange line), Py-2-SWNT (red line) and Py-ref (green line) in DMF recorded after excitation at 340 nm. The concentrations of the pyrene moieties of Py-1-SWNT, Py-2-SWNT, and Py-ref were adjusted to afford an absorbance of 0.03 at 340 nm. The inset shows the low emission intensity portion of the spectra. (**b–d**) Femtosecond TA spectra of (**b**) PhI-SWNT, (**c**) Py-1-SWNT, and (**d**) Py-2-SWNT in DMF. Spectra recorded using various time delays between 0.1 and 30 ps are shown. The excitation wavelength used was 350 nm. (**e**) A schematic charge separation mechanism. HOMO and LUMO energy levels of 1-phenylpyrene were determined based on the first oxidation and reduction potentials as measured using cyclic voltammetry. The HOMO level of the pyrene dimer in Py-1-SWNT is considered higher than that of the pyrene monomer by 0.30 V because the stabilization energy of pyrene dimer radical cation is reported to be ca. 0.3 eV^29^. The LUMO level of the pyrene dimer was estimated by adding the optical bandgap energy to the HOMO. Note here that the optical bandgap of the pyrene dimer is 2.5 eV, which was determined according to the absorption edge of the pyrene dimer moiety shown in Fig. 2. The energy levels of the lowest unoccupied conduction band (c_1_) of the SWNTs are taken from the literature^30^,^31^. The energy levels of the highest occupied valence band (v_1_) are estimated by adding the optical bandgap energies of the SWNTs (determined from the emission peaks) to the c_1_ level.

Py-1-SWNT, which contains dimeric pyrenes on SWNT, is anticipated to exhibit markedly different photodynamics than Py-2-SWNT, which contains monomeric pyrenes on SWNT. Steady-state fluorescence spectra reveal interactions between the pyrenyl groups and the pyrene–SWNT in Py-1-SWNT within the excited states (Fig. 7a). Upon the excitation of Py-1-SWNT at *λ*_ex_=340 nm, where the absorbance of the pyrene moiety was adjusted to be identical to that of Py-ref, the fluorescence of the pyrene moieties on the SWNTs was quenched almost quantitatively in comparison with Py-ref. No emission from the pyrene excimer at longer wavelengths (>450 nm)^18^ was detected. Py-2-SWNT also exhibited almost no fluorescence due to the pyrene moiety. The fluorescence lifetimes of Py-1-SWNT, Py-2-SWNT, PhI-SWNT and Py-ref were measured using the time-correlated single-photon counting technique by monitoring at *λ*_obs_=390 nm (*λ*_ex_=340 nm) (Supplementary Fig. 18). The fluorescence decay of Py-ref was analysed based on a single component with a lifetime (τ) of 30 ns. However, the fluorescence decay curves of Py-1-SWNT and Py-2-SWNT were fitted based on two fast components (*τ*=0.2–0.7 ns and *τ*=4–6 ns) and one slow component (*τ*=18–25 ns), results that are comparable to those obtained for PhI-SWNT. This result reveals that the observed weak fluorescence does not originate from the pyrene moieties on the SWNTs but from the functionalized SWNTs themselves^27^,^28^. Thus, fast relaxation pathways exist in which the excited pyrenes are quenched rapidly by the tethered SWNTs via short, rigid phenylene spacers at times that are shorter than those observable by these fluorescence lifetime measurements (<80 ps).

To further elucidate interaction in the excited states, the pump-probe transient absorption (TA) measurements were acquired (Fig. 7b–d and Supplementary Fig. 19). The TA spectra of PhI-SWNT exhibited almost no signals after a delay of 3 ps (Fig. 7b). Figure 7c shows the TA spectra of Py-1-SWNT at *λ*_ex_=350 nm with an absorption ratio of Py:SWNT=3:7. In addition to the positive signal observed in the <600 nm region at 0.1 ps, which reflects the formation of the SWNT excited state, TA spectra with longer time delays of up to 30 ps revealed characteristic positive bands at 500, 550 and 620 nm and negative bands at 580 and 670 nm. These spectral features suggest the evolution of a new transient species involving SWNT and the dimeric pyrenes. In contrast, the TA spectra of Py-2-SWNT are nearly identical to those of PhI-SWNT. Specifically, the pyrene-excited singlet state (^1^Py*) in Py-2-SWNT is rapidly quenched by the SWNT, possibly generating the SWNT excited state via energy transfer (EN) within the time resolution of the measurement system (∼0.15 ps). These significant differences in the TA of Py-1-SWNT and Py-2-SWNT might result from electron transfer (ET) from the pyrene dimer to the SWNT excited state solely in Py-1-SWNT, thereby yielding the oxidized pyrene dimer and the reduced SWNTs as illustrated in Fig. 7e (refs 29, 30, 31). Owing to the π–π interaction between pyrenes, the ionization potential of the pyrene dimer becomes lower than that of the pyrene monomer^29^. The rise in the HOMO level allows ET from the dimeric pyrenes to the excited SWNTs in Py-1-SWNT, whereas ET from the monomeric pyrenes remains energetically unfavourable.

**Figure 8:**
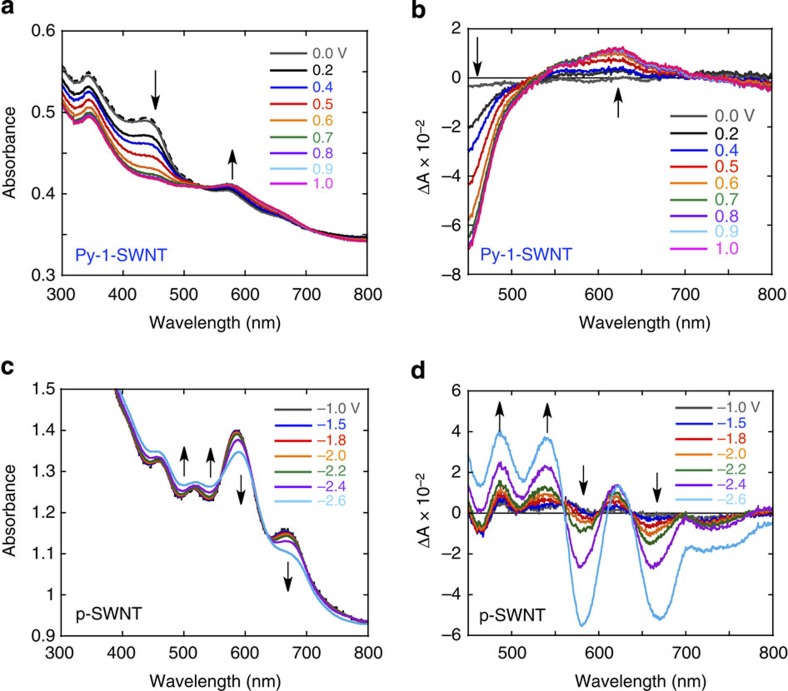
Spectroelectrochemistry. (**a**) UV-visible absorption spectral changes observed during the electrochemical oxidation of Py-1-SWNT by applying a potential from 0 to 1.0 V versus Ag/AgNO_3_ in DMF. The absorption spectrum of Py-1-SWNT recorded in the absence of an applied potential is shown by the dotted black line. (**b**) Differential absorption (ΔA) spectra of Py-1-SWNT with respect to oxidation. The reference spectrum corresponds to the absorption spectrum recorded in the absence of an applied potential. (**c**) UV-visible absorption spectral changes observed during the electrochemical reduction of p-SWNT under an applied potential of –1.0 to –2.6 V versus Ag/AgNO_3_ in DMF. The absorption spectrum of p-SWNT recorded in the absence of an applied potential is also shown by the dotted black line. (**d**) Differential absorption spectra of p-SWNT with respect to reduction. The reference spectrum corresponds to the absorption recorded in the absence of an applied potential. All measurements were conducted in DMF using Bu_4_NPF_6_ (20 mM) as an electrolyte.

To verify the photodynamic mechanism illustrated in Fig. 7e (the formation of the oxidized pyrene dimer and the reduced SWNTs) the spectral features of the pyrene dimer upon oxidation and those of the SWNTs upon reduction were characterized electrochemically (Fig. 8). Upon applying a potential of 0 to 1.0 V versus Ag/AgNO_3_, the absorption band at 450 nm in the spectrum of Py-1-SWNT was gradually lost, revealing that the pyrene dimer moieties rather than the SWNTs are predominantly oxidized (Fig. 8a). The differential absorption spectra exhibited a broad, weak positive signal in the 550–700 nm region and strong bleaching at <500 nm (Fig. 8b). However, the reduction of Py-1-SWNT led to no apparent spectral changes for a potential of up to –1.2 V versus Ag/AgNO_3_ due to the undesirable aggregation. Once these aggregates were formed, Py-1-SWNT did not redisperse in DMF even after the applied potential was reduced back to 0 V versus Ag/AgNO_3_. According to the energy diagram (Fig. 7e), the SWNT moiety is reduced before the pyrene dimer. These results indicate that the interaction between the neutral pyrene dimers and the reduced SWNTs caused the pyrene moieties to decompose. To characterize the absorption spectrum of the reduced SWNTs, we performed the spectroelectrochemical measurements for p-SWNT dispersed in DMF (Fig. 8c,d). The differential absorption spectra presented in Fig. 8d show positive peaks at ∼490, 540 and 620 nm and bleaching centred at 580 and 670 nm. In these measurements, the spectral features were reversibly transformed to those of the neutral form upon applying 0 V versus Ag/AgNO_3_. Thus, the spectral shape observed in the TA of Py-1-SWNT (Fig. 7c) is reproduced well by the sum of the spectra of the oxidized pyrene dimers and the reduced p-SWNTs, a finding that unambiguously confirms ET from the dimeric pyrenes to the excited SWNTs in Py-1-SWNT. Although ET from the singlet excited states of the pyrene monomer and dimer to SWNTs is also possible considering their LUMO energy levels, these states might be rapidly quenched by the SWNTs via EN to generate the SWNT excited state due to the full overlap between the emissions of the pyrene monomer and dimer and the absorptions of the SWNTs. The occurrence of ultrafast EN is consistent with the efficient emission quenching of the pyrene monomer and dimer (Fig. 7a). Overall, the difference in the molecular structures obtained for Py-1-SWNT and Py-2-SWNT accords well with the photodynamics results.

## Discussion

Selective formation of the dimeric and monomeric π-aromatic units on an sp^2^ carbon network of SWNTs was achieved using one- and two-step methods (direct addition of the PP radical onto the SWNT sidewall (Py-1-SWNT) and Suzuki coupling between PhI-functionalized SWNT and pyrene boronic ester (Py-2-SWNT)). Py-2-SWNT presented a monomeric pyrene located on the SWNT sidewall, whereas dimeric pyrenes were formed on the SWNT sidewalls of Py-1-SWNT, as demonstrated both theoretically and experimentally. In particular, we successfully visualized for the first time the linking of small polycyclic planar molecules onto the outside of nanocarbon scaffolds at a single-molecule level using the HR-TEM technique. Reflecting the unique orientation and separation distances between the two pyrene moieties, Py-1-SWNT exhibited an additional distinct band in the absorption spectrum and a photoinduced charge separation in the TA spectrum, which is in marked contrast with Py-2-SWNT. The present methodology used to form and probe molecular dimers on SWNT is applicable to the study of any organic molecules that contain aryl groups, including π-conjugated small molecules and oligomers. Moreover, the orientation and separation distances between the two molecules can be controlled, depending on the molecular interactions and reactivity to the sidewall of the SWNT, thereby yielding a variety of optical and photophysical properties. Thus, this methodology can be used to provide basic information regarding the close structure–property relationships existing within dimeric molecules of interest.

## Methods

### Instruments

^1^H and ^13^C NMR spectra were recorded using a JEOL JNM-EX400 NMR spectrometer. Attenuated total reflectance (ATR) FT-IR spectra were recorded using a Thermo Fisher Scientific Nicolet 6700 FT-IR. High-resolution mass spectra (HRMS) were obtained using a JEOL JMS-T100CS instrument. X-ray photoelectron spectroscopy (XPS) was performed using an ULVAC-PHI MT-5500 system with Mg Kα. Resonance Raman spectra were measured using a Horiba Jobin Yvon LabRAM ARAMIS spectrometer equipped with 2.33 eV (532 nm) and 1.96 eV (633 nm) lasers. TGA measurements were conducted using a SHIMADZU TGA-50 system under flowing nitrogen at a scan rate of 5 °C min^–1^. AFM analyses were performed using an Asylum Technology MFP-3D-SA system operating in the AC mode. Dispersions in DMF were spin-coated onto freshly cleaved mica at 1,500 r.p.m. UV-visible-near infrared (NIR) absorption spectra of the solutions and films were measured using a Perkin-Elmer Lambda 900 UV/vis/NIR spectrometer. Steady-state fluorescence spectra were recorded using a HORIBA SPEX Fluoromax-3 spectrofluorometer. A time-correlated single photon counting (TCSPC) method was employed to measure the fluorescence lifetime using a HORIBA SPEX Fluorolog-3+TCSPC instrument. Pump-probe measurements were performed using Libra-F (Coherent) and Topas-C (Light Conversion) laser systems to produce pump and probe pulses and an ExciPro (CDP) measurement system to record the time-resolved absorption spectra^32^. Typically, the time resolution of the instrument was 150 fs (FWHM). Electrochemical measurements were performed at room temperature using a CH Instruments model 660A electrochemical workstation equipped with a glassy carbon working electrode, a platinum wire counter electrode and an Ag/Ag^+^ (0.01 M AgNO_3_, 0.1 M Bu_4_NPF_6_ (MeCN)) reference electrode. The potentials were calibrated using ferrocene/ferrocenium (Fc/Fc^+^).

### Materials

All solvents and chemicals used were of reagent grade quality and used as purchased without further purification. SWNTs produced using the CoMoCAT method (SouthWest Nano Technologies) were purchased from Sigma-Aldrich Co.

### Theoretical studies

Geometry optimizations of the *p*-iodinephenyl group attached to nanotubes with a chiral index of (6,6) were performed using DFT/RB3LYP with an effective core potential (ECP) basis set combination LanL2DZ at iodine^33^ as implemented in the Gaussian 03 program package^34^. The elements C and H were described using the standard basis set 3-21G*. Geometry optimizations of pyrene-functionalized SWNTs were conducted using the B97D functional, which was previously parameterized for dispersion interactions^35^. The B97D functional contributes significantly to the π-π interaction, that is, stacked pyrene rings. Finite-length nanotubes terminated by H atoms with chiral indices of (9,4) and (6,6) were employed as the models. At the final geometries, the vertical singlet–singlet electronic transition energies of the pyrenyl moieties were calculated at the same TD-RB3LYP/6-31G levels using TD-DFT after omitting the SWNT scaffolds for clarity. All calculations were performed in the gas phase.

### HR-TEM Measurements

Specimens were prepared by dispersing the pyrene-functionalized SWNTs in ethanol and then dropping the resulting dispersions onto copper micro-grids coated with perforated amorphous carbon films. HR-TEM measurements were performed using a JEOL JEM-2100F microscope equipped with Delta spherical aberration (Cs) correctors at an electron acceleration voltage of 60 kV (ref. 36) and using a JEOL JEM-2010F microscope equipped with a CEOS Cs corrector at 120 kV (ref. 37). HR-TEM images were recorded using CCD cameras (Gatan, model 894) installed in these microscopes at a rate of 1 frame s^−1^. TEM image simulation was performed using SimulaTEM software^38^.

### 4-(1-Pyrenyl)benzenediazonium tetrafluoroborate

A solution of 1-(4-aminophenyl)pyrene^39^ (3.2 g, 11 mmol) in DME (25 ml) was slowly added with agitation to a round-bottomed flask charged with a solution of BF_3_·OEt_2_ (12.5 ml, 99 mmol) in 1,2-dimethoxyethane (DME, 8 ml) at −5 °C. Then, *t*-BuONO (6.5 ml, 55 mmol) was added dropwise over at least 30 min using a syringe. A precipitate formed during the addition. The reaction mixture was stirred at −5 °C for 1 h after the *t*-BuONO addition was complete. The reaction mixture was stirred and the brine/ice bath in which the reaction was conducted was allowed to warm to 0 °C. The reaction mixture was then filtered to recover the resulting black solid, which was then washed with DME three times to afford the diazonium salt as a dark-brown powder to yield the target compound (3.0 g, 70%). M.p. 110–113 °C; ^1^H NMR (CD_3_OD, 300 MHz): *δ* 8.82 (*d*, 2H, *J*=9.2 Hz), 8.39 (*s*, 1H), 8.37 (*s*, 1H), 8.35 (*s*, 1H), 8.31 (*d*, 2H, *J*=9.2 Hz), 8.24 (*s*, 1H), 8.21 (*s*, 1H), 8.14–8.07 (*m*, 4H). ^13^C NMR *δ*_C_ (CD_3_CN, 100.4 MHz): 154.71, 133.27, 132.56, 132.18, 132.07, 130.87, 130.23, 128.75, 128.58, 127.65, 127.24, 126.81, 126.42, 125.97, 125.50, 124.66, 124.10, 123.69, 122.93, 111.52. IR (ATR): 3,102, 2,272, 1,574, 1,505, 1,413, 1,308, 1,242, 1,191, 1,034, 766, 724, 682, 585, 554, 519, 454 cm^–1^. HRMS (ESI+, *m*/*z*): calcd. for [C_22_H_13_N_2_]^+^, 305.1079; found 305.1073.

### Py-1-SWNT

4-(1-Pyrenyl)benzenediazonium tetrafluoroborate (1.7 g, 4.2 mmol) was added to a surfactant (sodium dodecylbenzenesulfonate, SDBS)-coated dispersion of p-SWNT^40^ (5.0 mg). The reaction mixture was stirred for 1 h at room temperature, and then poured into acetone to precipitate a black powder. After filtration through a PTFE membrane with an average pore size of 0.1 μm, the black precipitate was washed with water and acetone to remove the SDBS and excess diazonium salt. The black powder was redispersed in DMF and centrifuged at 14,000 *g* for 10 min. Next, the supernatant was filtered and washed thoroughly with DMF on the PTFE membrane three times. The resulting black cake was suspended in DMF and then filtered and washed with DMF. The resulting solid was dried under a vacuum overnight to yield 2.3 mg of Py-1-SWNT.

### PhI-SWNT

4-Iodobenzenediazonium tetrafluoroborate^41^ (1.3 g, 4.2 mmol) was added to a SDBS-coated dispersion of p-SWNT^40^ (5.0 mg) and then stirred at room temperature for 1 h. The reaction mixture was diluted with acetone and filtered through a PTFE membrane with an average pore size of 0.1 μm. The black material on the filter paper was washed with acetone, methanol and DMF to efficiently remove SDBS and excess unreacted diazonium salt, and the mixture was then diluted with DMF and filtered through a PTFE membrane. The resulting black solid was redispersed in DMF and centrifuged at 14,000 *g* for 10 min. Next, the supernatant was filtered and washed thoroughly with DMF on the PTFE membrane three times. The resulting solid was dried under a vacuum overnight to yield 5.8 mg of PhI-SWNT.

### Py-2-SWNT

A flask was charged with PhI-SWNT (6.0 mg) and DMF (5 ml). After a bath-type sonication for 10 min at room temperature, Cs_2_CO_3_ (0.2 g, 0.6 mmol) and 1-pyreneboronic acid (20 mg, 0.08 mmol) were added. The resulting was then degassed via three cycles of freeze-pump-thaw and recharged with argon. Pd(PPh_3_)_4_ (10 mg) was added and the reaction mixture was then stirred for 24 h at 105 °C under an argon atmosphere. After cooling to room temperature, the mixture was diluted with THF, sonicated for 10 min and filtered through a PTFE membrane. The product was washed thoroughly with THF, methanol, water, methanol and THF to remove excess pyrene. The resulting black cake was redispersed in DMF and centrifuged at 14,000 *g* for 10 min. Next, the supernatant was filtered and washed thoroughly with DMF on the PTFE membrane three times. The resulting solid was dried under a vacuum overnight to yield 2.6 mg of Py-2-SWNT.

## Additional information

**How to cite this article:** Umeyama, T. *et al*. Molecular interactions on single-walled carbon nanotubes revealed by high-resolution transmission microscopy. *Nat. Commun.* 6:7732 doi: 10.1038/ncomms8732 (2015).

## Supplementary Material

Supplementary Information Supplementary Figures 1-19 and Supplementary References
