# Supplementary material for: Molecular interactions on single-walled carbon nanotubes revealed by high-resolution transmission microscopy
Source: Nat Commun. 2015 Jul 15;6:7732. doi: 10.1038/ncomms8732 (PMC4518305; doi:10.1038/ncomms8732)
Supplement: Supplementary Information — Supplementary Figures 1-19 and Supplementary References [file ncomms8732-s1.pdf]

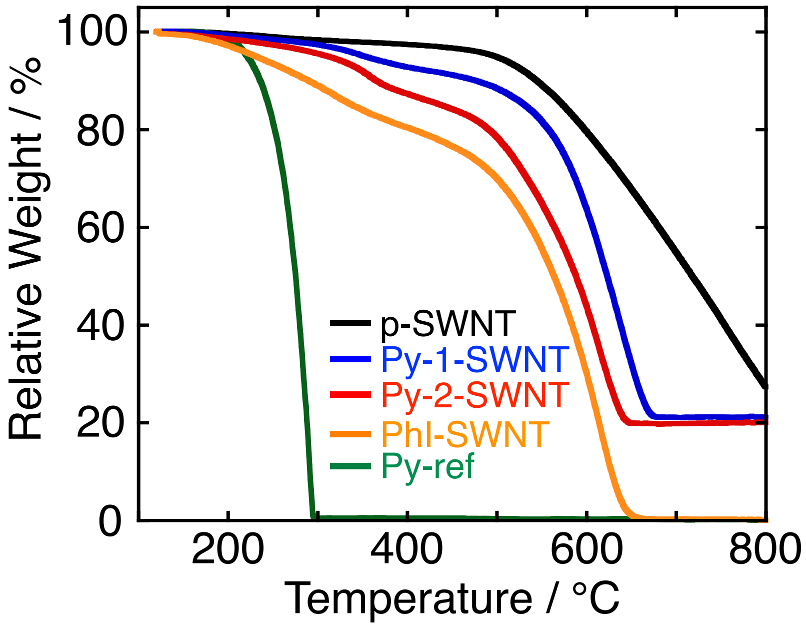

Supplementary Figure 1 | Thermogravimetric analyses (TGA). p-SWNT (black line), Py-1-SWNT (blue line), Py-2-SWNT (red line), PhI-SWNT (orange line), and 1-phenylpyrene (Py-ref, green line). The analyses were performed under nitrogen with a heating rate of 5 deg min^{-1}. The residues of Py-1-SWNT and Py-2-SWNT after the TGA measurements were found to consist of only carbon atoms by XPS measurements.

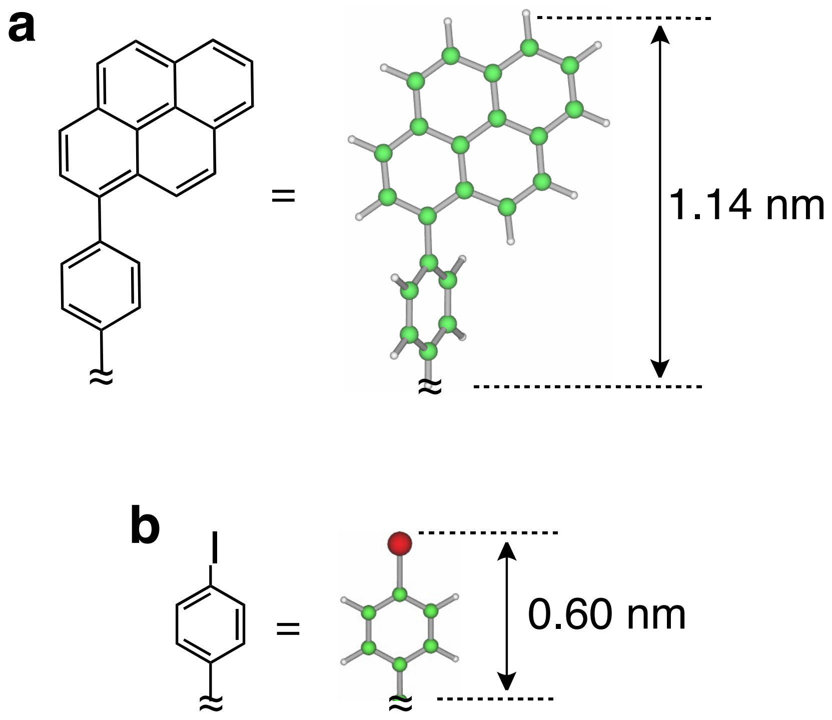

Supplementary Figure 2 | Chemical structures and sizes of the substituents. a, PP group optimized using RB3LYP functional and 6-31G basis set and **b**, 4-iodophenyl group obtained from RB3LYP functional and 3-21G* and LanL2DZ mixed basis set implemented in Gaussian03 program package.

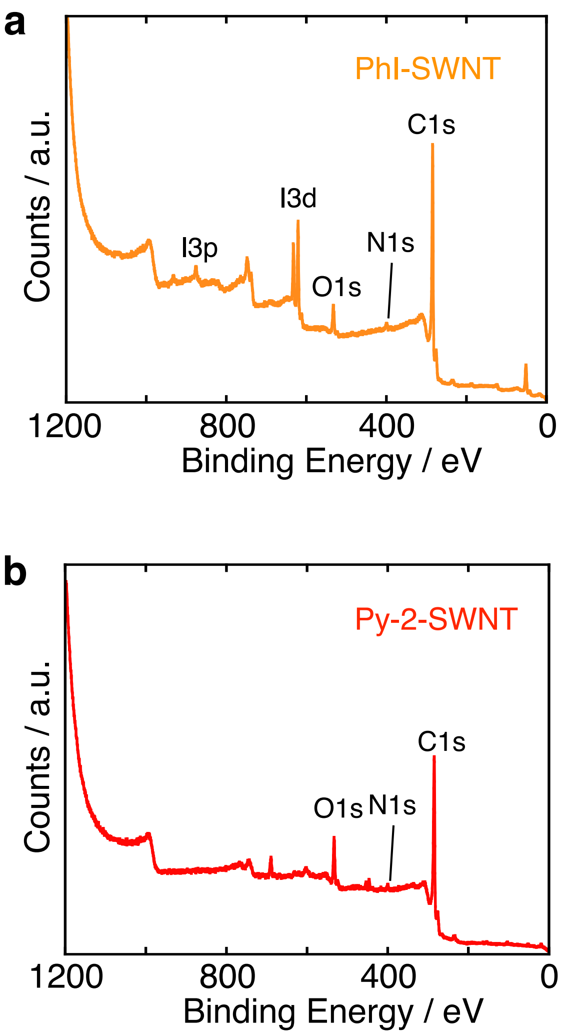

Supplementary Figure 3 | XPS survey scans. a, PhI-SWNT and **b**, Py-2-SWNT.

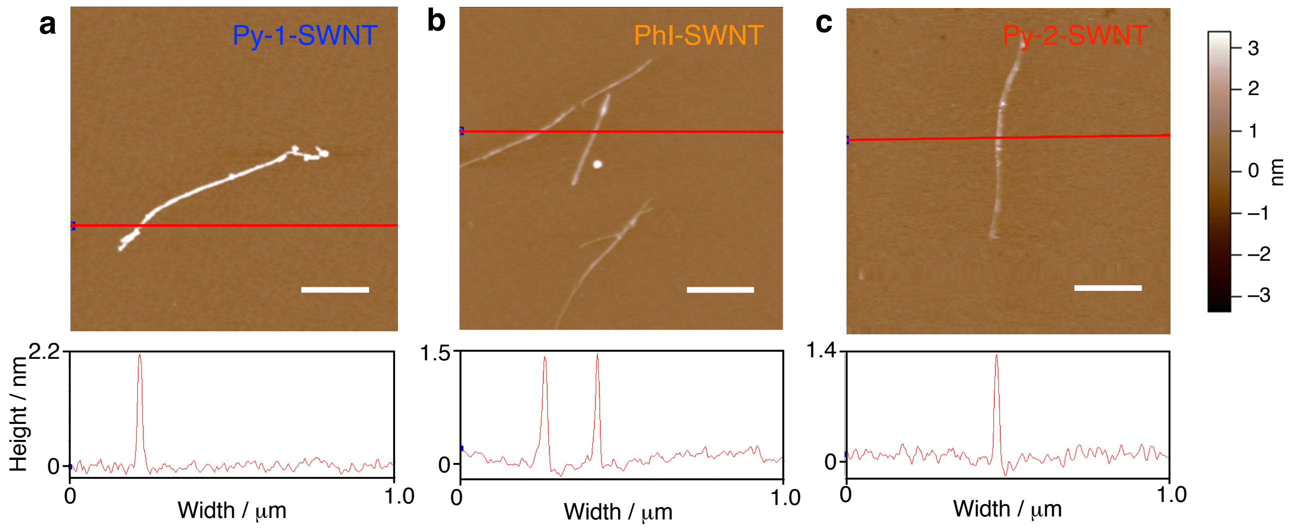

Supplementary Figure 4 l AFM images with section profiles. a, Py-1-SWNT, **b**, PhI-SWNT, and **c**, Py-2-SWNT spin coated on mica from DMF dispersion. The scale bars are 200 nm. The color scales represent the height topography, with light and dark representing the highest and lowest features, respectively. All of the images demonstrate the efficient debundling of SWNTs by the functionalization to isolated nanotubes or bundles of only a few nanotubes.

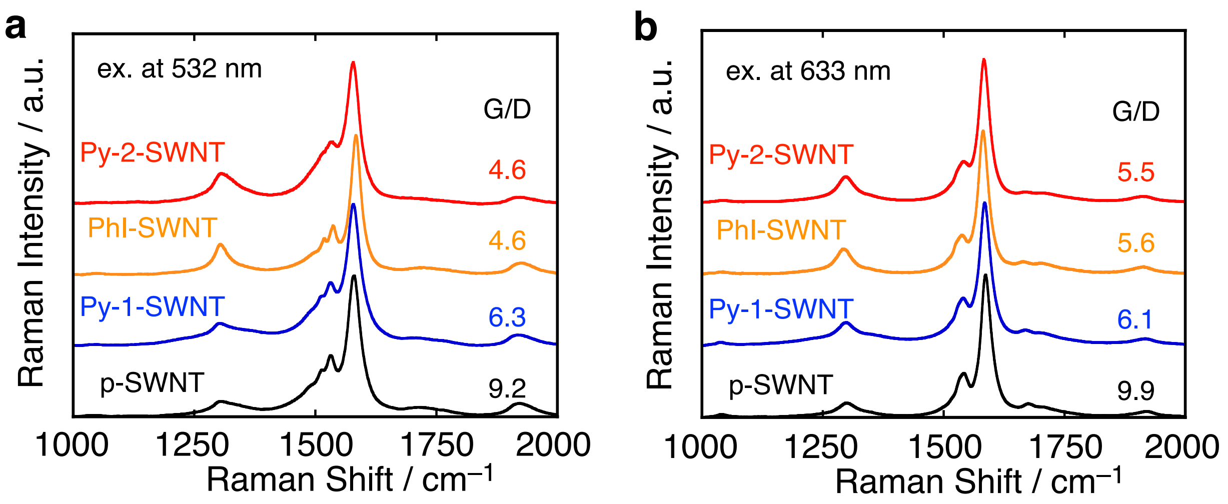

Supplementary Figure 5 I Resonant Raman spectra. p-SWNT (black line), Py-1-SWNT (blue line), PhI-SWNT (orange line), and Py-2-SWNT (red line) with excitation wavelengths of **a**, 532 nm and **b**, 633 nm. The relative peak intensities of tangential mode (G-band) around 1580 cm^{-1} and of disorder mode (D-band) around 1300 cm^{-1} (G/D ratio) are also shown. The G/D ratio reflects the relative amounts of sp^3 carbon, and is used to determine the degree of sidewall functionalization.[1] The G/D ratios of PhI-SWNT (4.6 and 5.6 by excitation at 532 and 633 nm, respectively) are much smaller than those of p-SWNT before functionalization (9.2 and 9.9), which is reasonable considering that the iodophenyl functionalization increases the defect sites on the sidewall of SWNT. The G/D ratios of Py-2-SWNT (4.6 and 5.5) are largely equal to those of PhI-SWNT. It means that the pyrene reagent reacted with the iodophenyl group solely and did not attack the sidewall of SWNT during the second step reaction. Meanwhile, the G/D ratios of Py-1-SWNT (6.3 and 6.1) are larger than those of PhI-SWNT and Py-2-SWNT, reflecting the lower total covalent functionalization ratio.

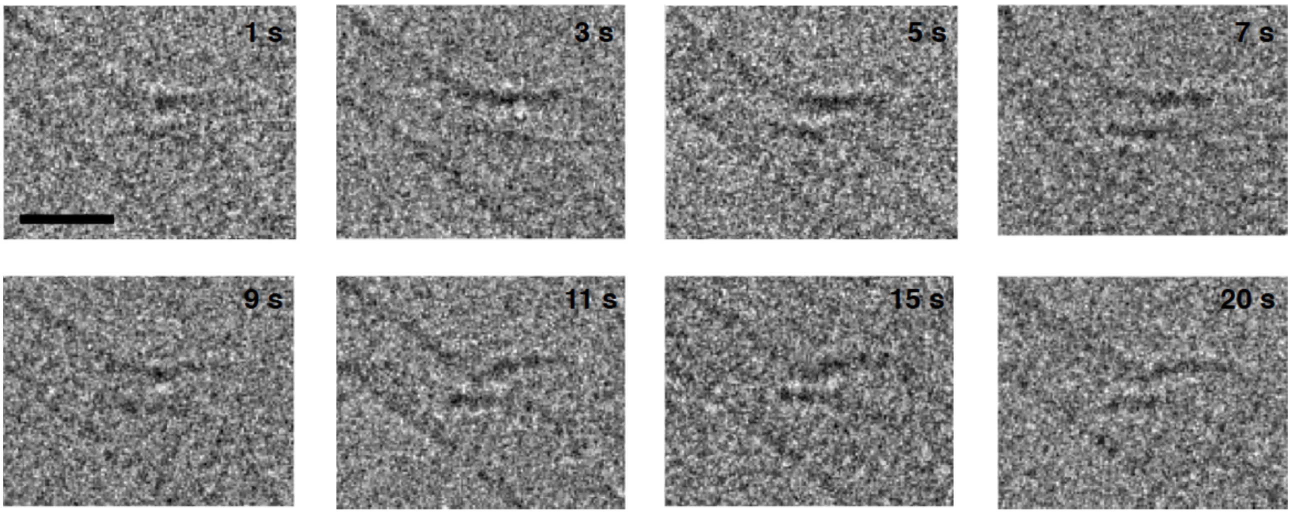

Supplementary Figure 6 l Sequential HR-TEM images of Py-1-SWNT. Scale bar is 1 nm. The images show stable stacking of a pyrene dimer over a period of 20 s.

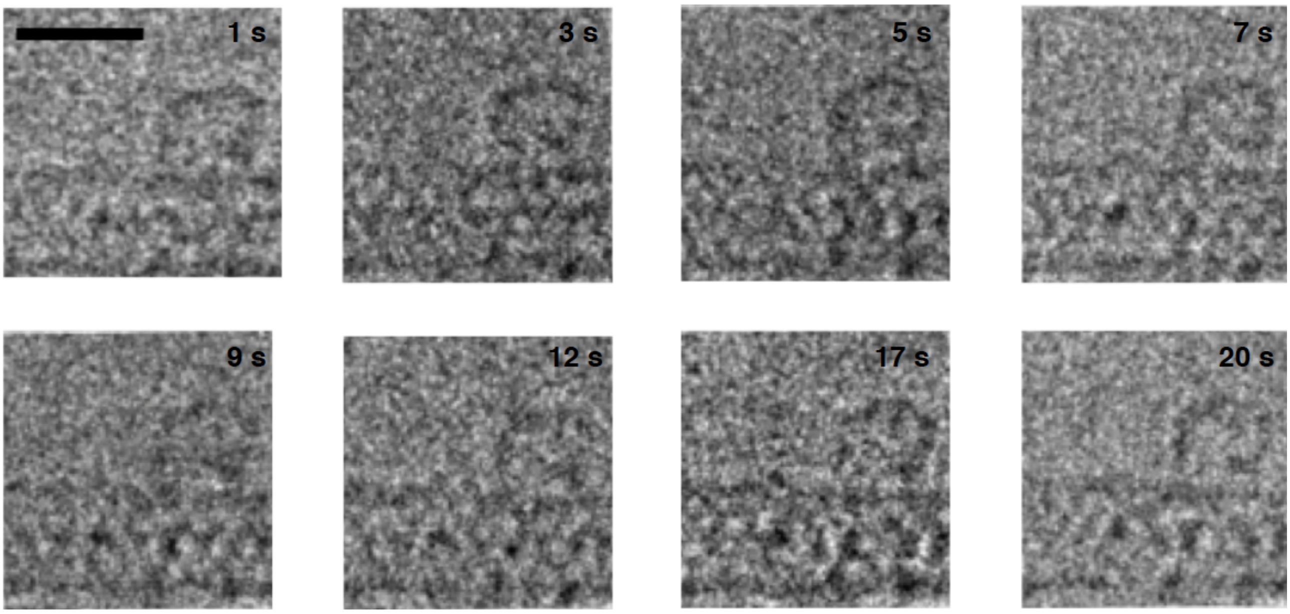

Supplementary Figure 7 l Sequential HR-TEM images of Py-2-SWNT. Scale bar is 1 nm. The images show a standing monomeric pyrene over a period of 20 s.

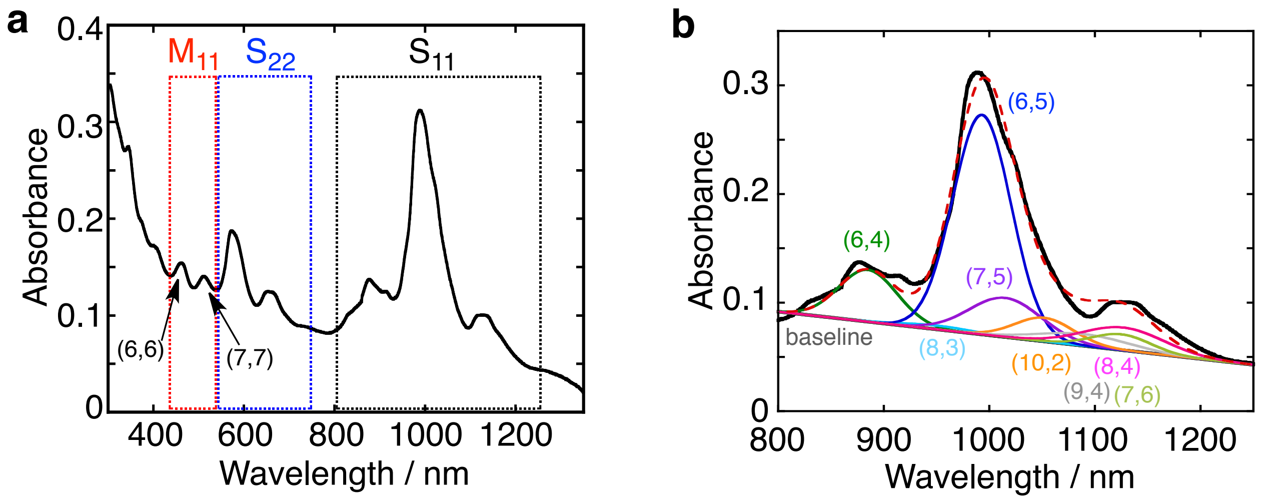

Supplementary Figure 8 | Absorption spectrum and chirality composition estimation of p-SWNT. a, UV-vis-NIR absorption spectra of p-SWNT dispersed in water with 1 wt% SDBS. The dotted lines show the absorption regions of SWNTs arising from the electronic transition between the first van Hove singularities of semiconducting SWNTs (S_{11}, black), the second van Hove singularities of semiconducting SWNTs (S_{22}, blue), and the first van Hove singularities of metallic SWNTs (M_{11}, red). The peaks at 463 nm and 513 nm in the M_{11} region can be assigned to the absorptions of metallic (6,6) and (7,7) SWNTs, respectively.[2] According to the supplier data, the content of metallic SWNTs is < 5%. **b**, Estimation of the chirality composition of p-SWNT by peak fitting of the S_{11} absorptions. The peaks are labeled by the corresponding chiral indices with the same color. The red dotted line shows the sum of all the peaks and baseline. The composition of each (*n,m*) was calculated as the ratio of the area of the certain peak to the sum of all the peak areas (composition (n_1,m_1) = area $(n_1,m_1)/\Sigma$ area (n,m)),[3] resulting in 53% (6,5), 12% (6,4), 12% (7,5), 8% (8,4), 6% (10,2), 4% (9,4), 4% (7,6), and 1% (8,3). Note here that the content of metallic SWNTs is excluded from the calculation.

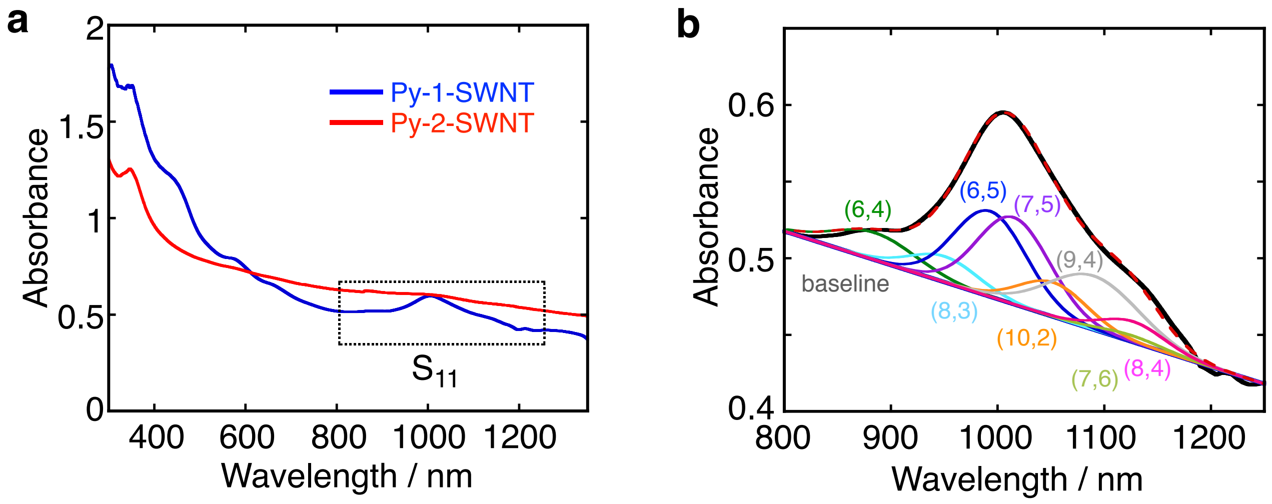

Supplementary Figure 9 | Absorption spectra of Py-1-SWNT and Py-2-SWNT and chirality composition estimation of Py-1-SWNT. **a**, UV-vis-NIR absorption spectra of Py-1-SWNT (blue line) and Py-2-SWNT (red line) in DMF. Note here that the concentrations are higher than those of the samples shown in Fig. 2 for the peak fitting of S_{11} absorptions. The absorption bands of the SWNT moieties in Py-2-SWNT are more significantly broadened than those of Py-1-SWNT due to the higher functionalization ratio, hindering the estimation of the chirality composition of Py-2-SWNT. **b**, Estimation of the chirality composition of Py-1-SWNT by peak fitting of the S_{11} absorptions as conducted in Supplementary Fig. 8, resulting in 25% (6,5), 9% (6,4), 25% (7,5), 5% (8,4), 9% (10,2), 19% (9,4), 1% (7,6), and 7% (8,3). SWNTs with chiral indices of (8,3), (7,5), and (9,4) were enriched by the aryl addition reaction and purification procedures.

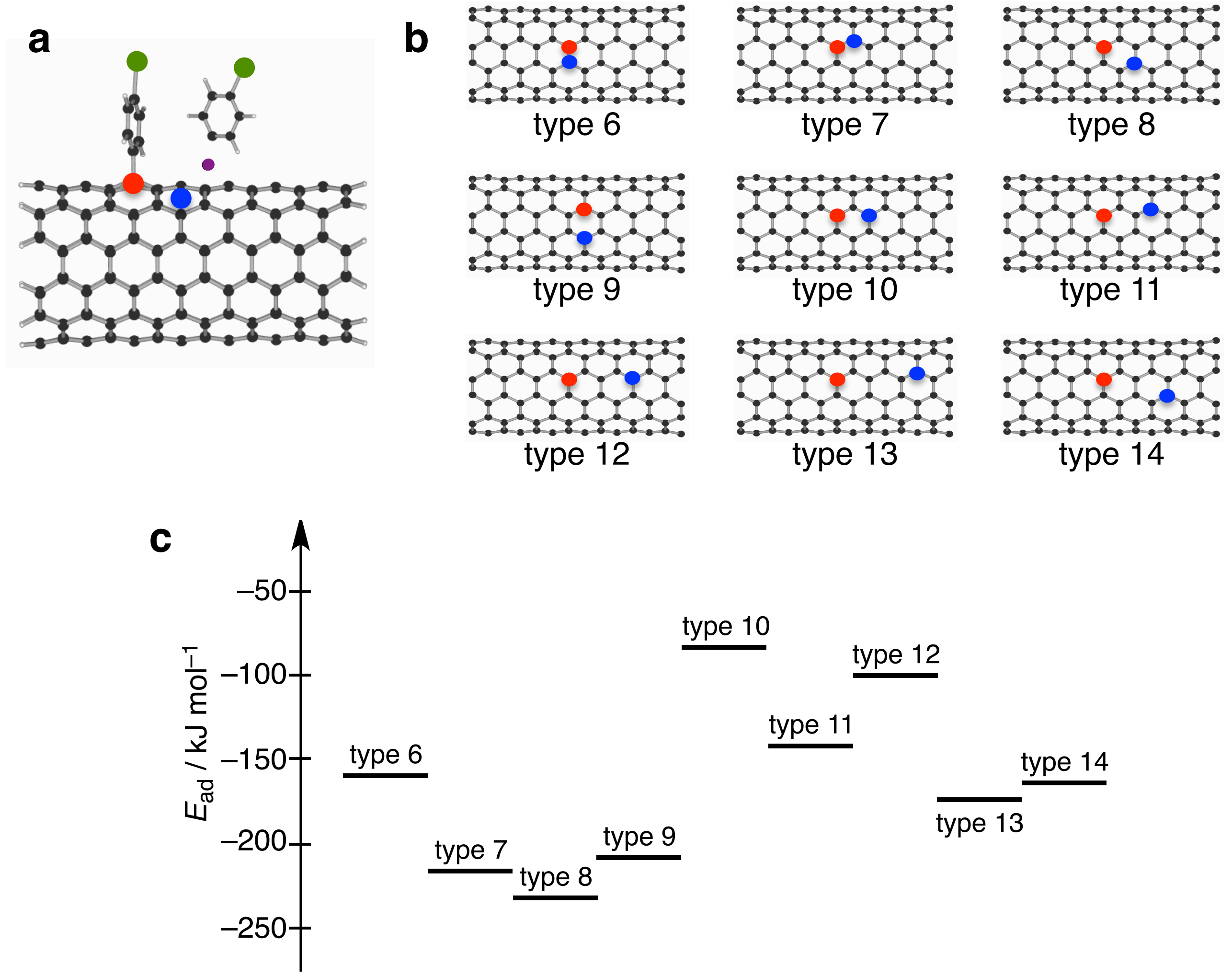

Supplementary Figure 10 | Second addition of PhI radical on (6,6)SWNT model. a, Schematic image. **b**, Different configurations for the second attachment of one PhI radical on the (6,6)SWNT model ($C_{168}H_{24}$). Red and blue circles represent the sites for the first and second (a purple dot in **a** denotes an unpaired electron) attachments, respectively. **c**, Energetics of different configurations for the second attachment of one PhI radical on the (6,6)SWNT model estimated by DFT calculations at RB3LYP/3-21G*+LanL2DZ level. The binding energy of the second PhI is determined by E_{ad} = *E*(2PhI/(6,6)SWNT) − *E*(PhI/(6,6)SWNT) −*E*(PhI). The energetically favored configurations (types 7-9) possess the two PhI groups at *para* and *ortho* positions in the same hexagon.

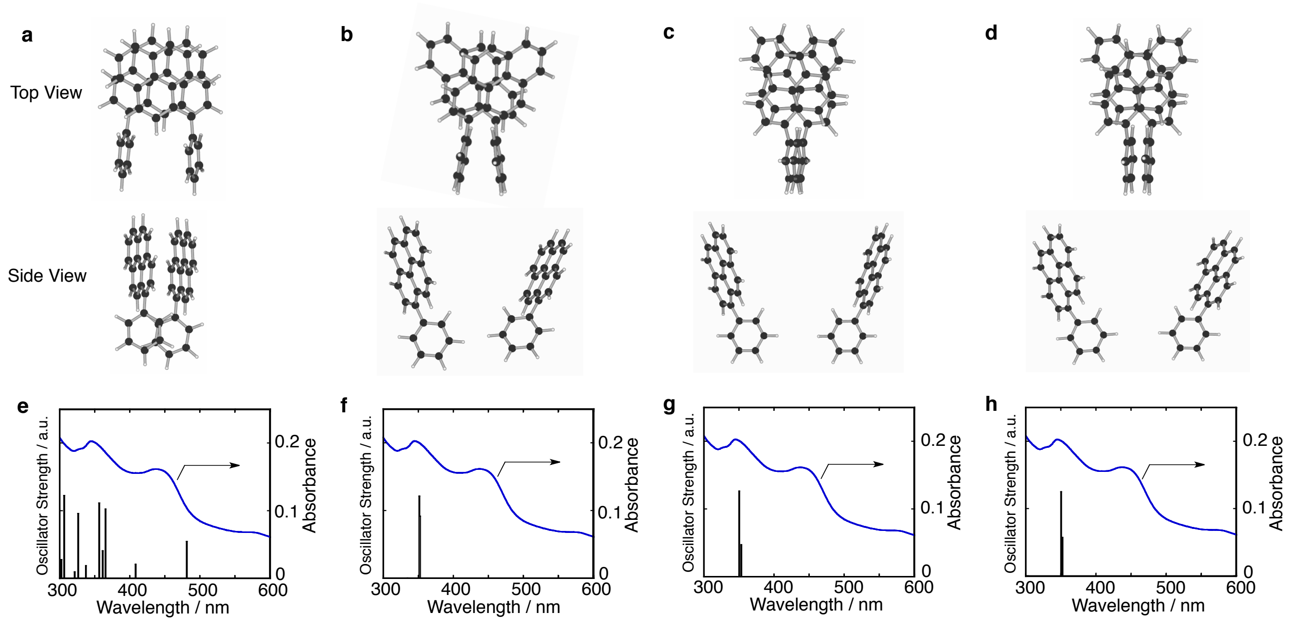

Supplementary Figure 11 l Structures and oscillator strength of PP dimer models. a-d, Optimized structures of the PP dimer taken from the Py-1-SWNT model with the configurations of (**a**) type 2, (**b**) type 3, (**c**) type 4, and (**d**) type 5 in Fig. 4. **e-h**, Oscillator strength vs. electronic transition energies for the PP dimer taken from the Py-1-SWNT model with the configurations of (**e**) type 2, (**f**) type 3, (**g**) type 4, and (**h**) type 5 in Fig. 4. The experimental UV-vis absorption spectrum of Py-1-SWNT as depicted in Fig. 2 is also shown for comparison. Note here that the optimized structure and oscillator strength of the PP dimer with the type 1 configuration are shown in Fig. 6.

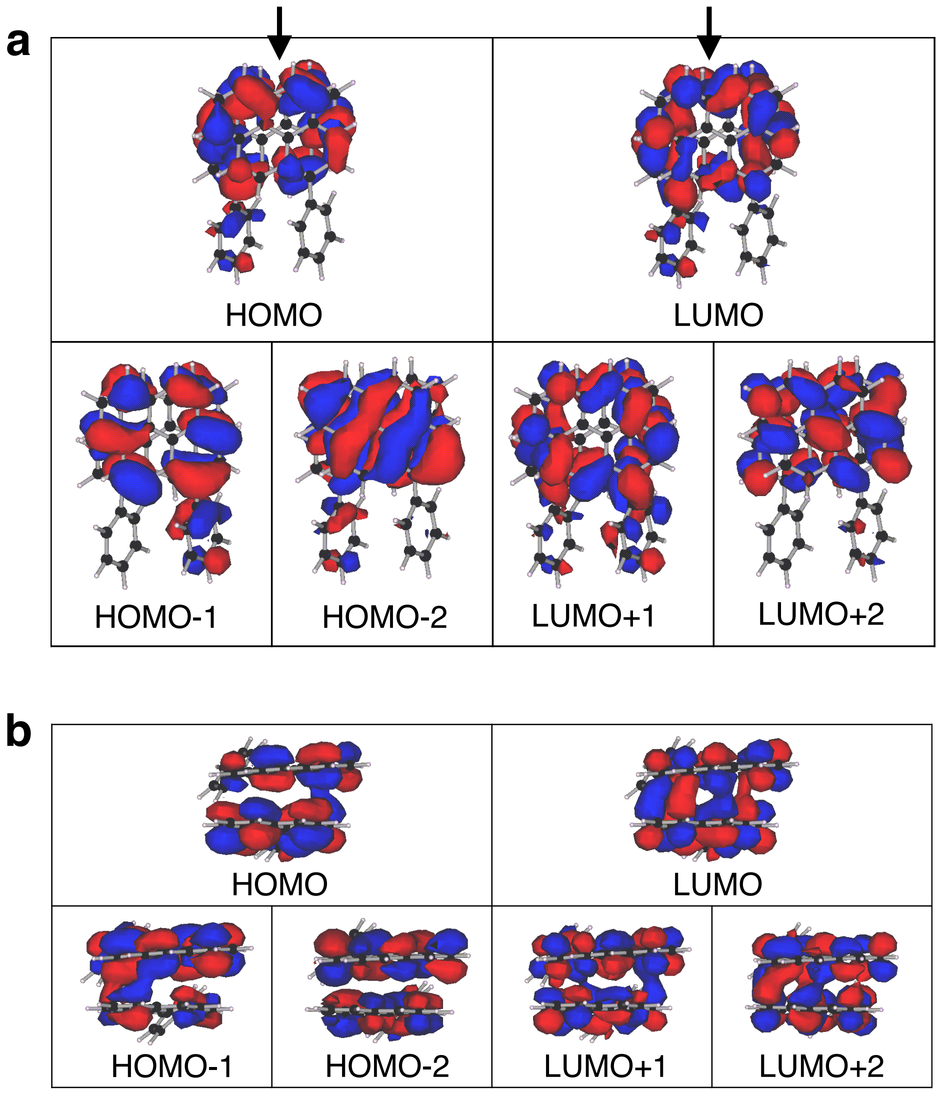

Supplementary Figure 12 | Orbital counter plots of PP dimer in Py-1-SWNT model with type 1 configuration. a, Top views and **b**, side views from the directions indicated by the arrows in **a**. The plots of the 3 highest occupied molecular orbitals and the 3 lowest unoccupied molecular orbitals are shown.

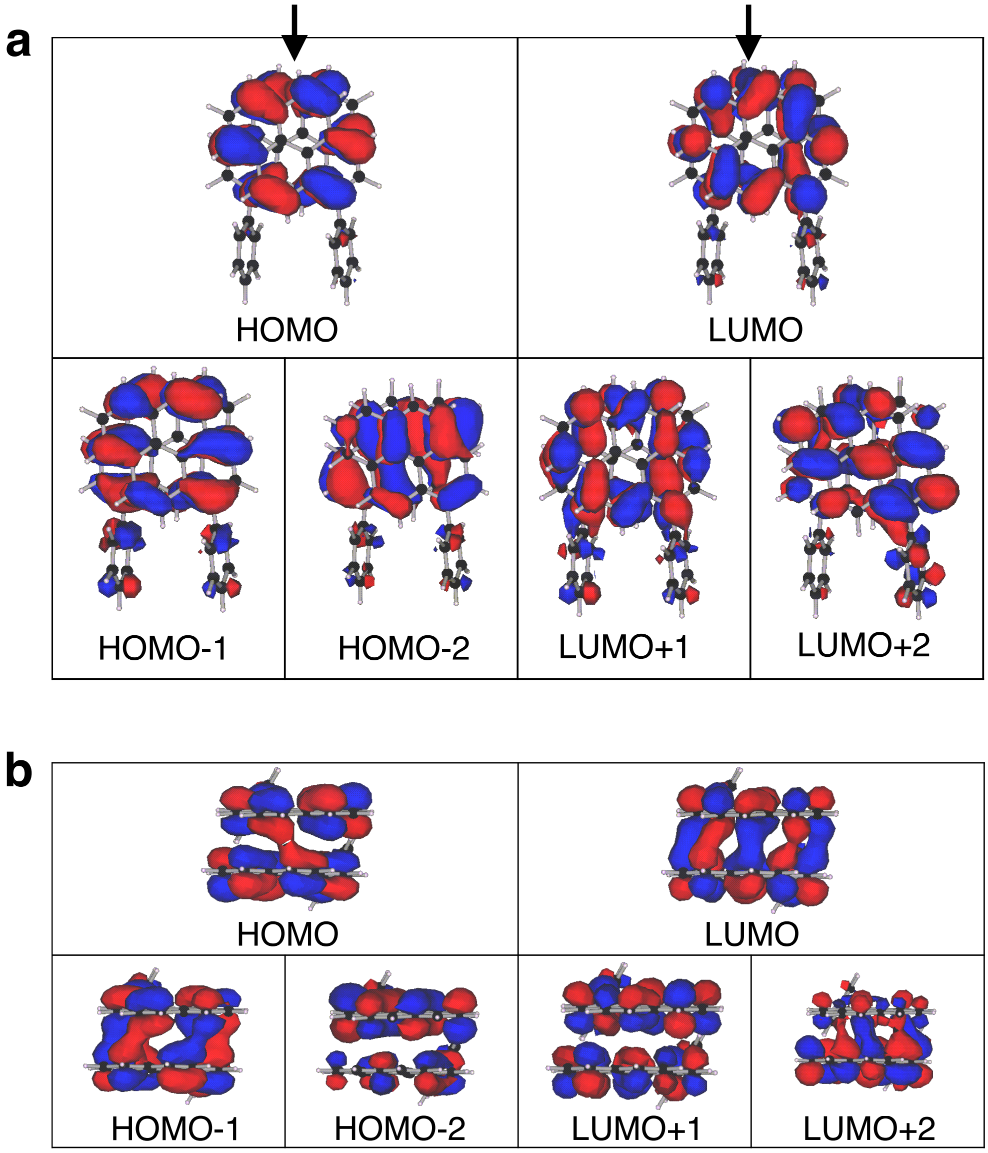

Supplementary Figure 13 l Orbital counter plots of PP dimer in Py-1-SWNT model with type 2 configuration. a, Top views and **b**, side views from the directions indicated by the arrows in **a**. The plots of the 3 highest occupied molecular orbitals and the 3 lowest unoccupied molecular orbitals are shown.

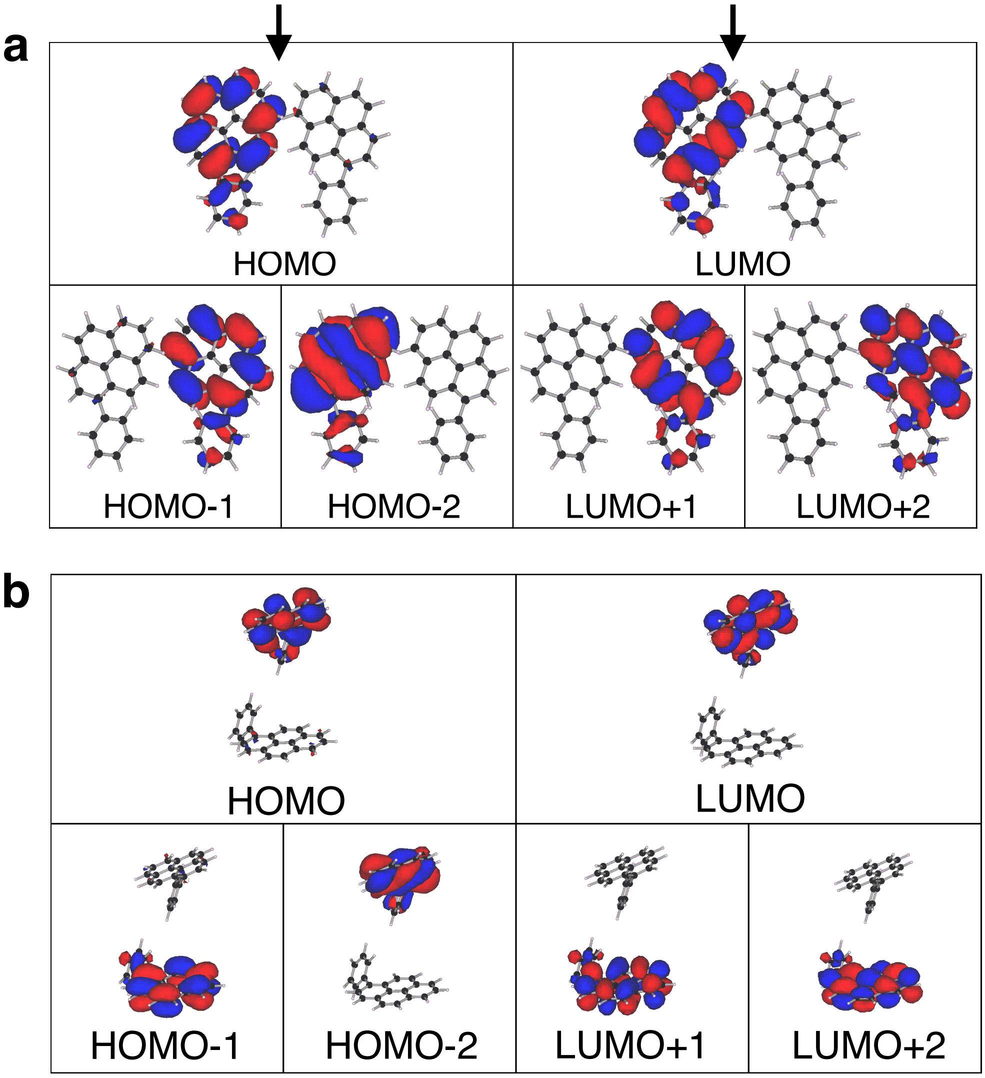

Supplementary Figure 14 l Orbital counter plots of PP dimer in Py-1-SWNT model with type 3 configuration. a, Top views and **b**, side views from the directions indicated by the arrows in **a**. The plots of the 3 highest occupied molecular orbitals and the 3 lowest unoccupied molecular orbitals are shown.

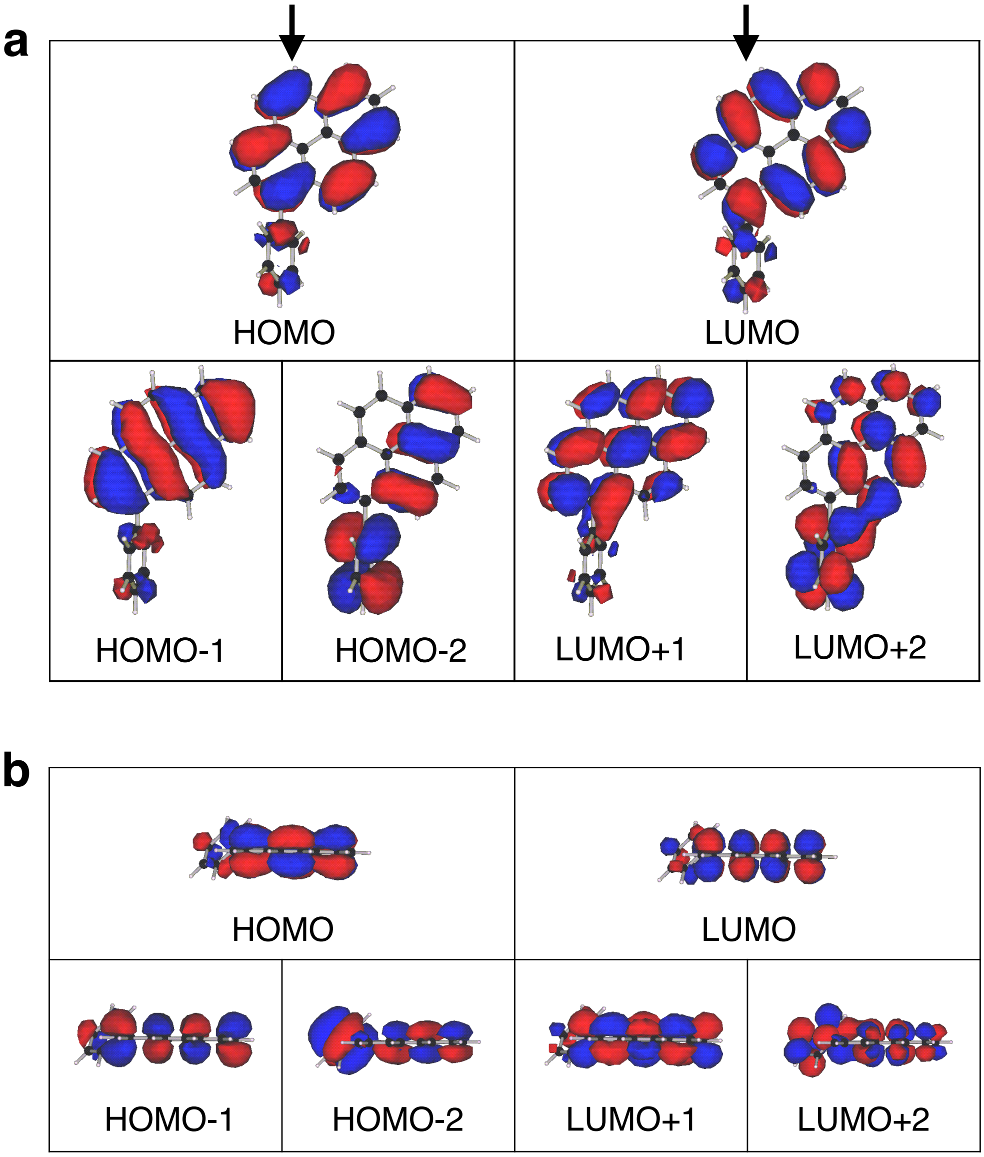

Supplementary Figure 15 | Orbital counter plots of Py-ref. a, Top views and **b**, side views from the directions indicated by the arrows in **a**. The plots of the 3 highest occupied molecular orbitals and the 3 lowest unoccupied molecular orbitals are shown.

a

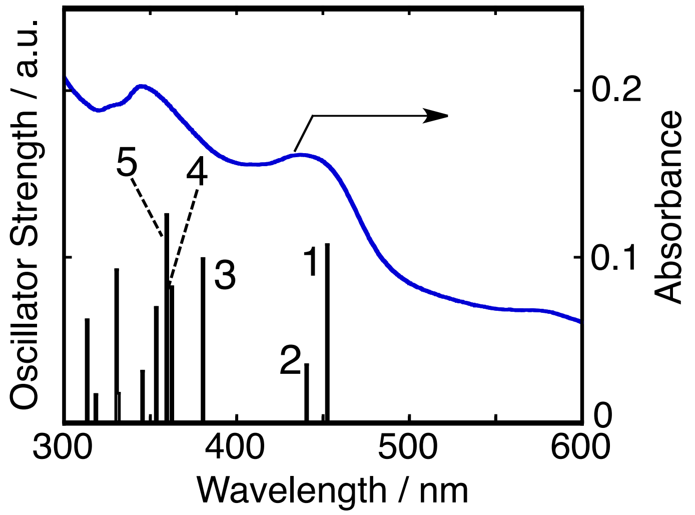

b

Transition	Excitation Energy eV	Excitation Energy nm	Oscillator Strength	Composition / %	
1	2.74	452.4	0.1034	HOMO-1 → LUMO	3.3
				HOMO-1 → LUMO+1	2.6
				HOMO → LUMO	87.4
				HOMO → LUMO+1	4.3
2	2.82	440.4	0.0334	HOMO-1 → LUMO	82.2
				HOMO-1 → LUMO+1	3.3
				HOMO → LUMO	4.5
				HOMO → LUMO+1	8.9
3	3.27	379.5	0.0952	HOMO-3 → LUMO	4.1
				HOMO-2 → LUMO	9.3
				HOMO-1 → LUMO	6.1
				HOMO-1 → LUMO+1	5.1
				HOMO → LUMO+1	69.9
4	3.43	361.7	0.0788	HOMO-3 → LUMO	4.6
				HOMO-2 → LUMO	33.0
				HOMO-1 → LUMO	3.3
				HOMO-1 → LUMO+1	41.7
				HOMO-1 → LUMO+2	4.7
				HOMO → LUMO+1	5.3
				HOMO → LUMO+2	2.5
5	3.45	359.1	0.1209	HOMO-3 → LUMO	2.7
				HOMO-2 → LUMO	31.1
				HOMO-2 → LUMO+1	2.5
				HOMO-1 → LUMO+1	24.9
				HOMO-1 → LUMO+2	10.1
				HOMO → LUMO	3.3
				HOMO → LUMO+2	18.2
				HOMO → LUMO+3	3.4

Supplementary Figure 16 | Absorption spectroscopic features of PP dimer in Py-1-SWNT model with type 1 configuration. a, Oscillator strength vs. electronic transition energies with the experimental absorption spectrum of Py-1-SWNT. The transition numbers in **b** are shown in **a**. **b**, Excitation energies, oscillator strengths, and compositions of the transitions. The transitions whose oscillator strengths are less than 2% are not included.

a

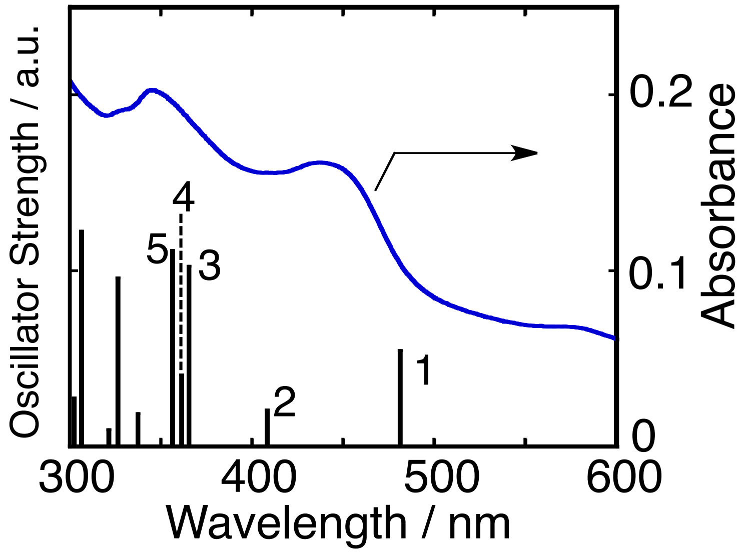

b

Transition	Excitation Energy eV	Excitation Energy nm	Oscillator Strength	Composition / %	
1	2.58	480.7	0.0437	HOMO → LUMO	97.3
2	3.04	408.0	0.0164	HOMO-1 → LUMO	42.9
				HOMO → LUMO+1	55.1
3	3.39	365.3	0.0821	HOMO-2 → LUMO	10.4
				HOMO-1 → LUMO	26.1
				HOMO → LUMO+1	16.3
				HOMO → LUMO+2	37.1
				HOMO → LUMO+3	4.8
4	3.43	361.1	0.0325	HOMO-2 → LUMO	15.3
				HOMO-1 → LUMO	11.3
				HOMO → LUMO+1	4.3
				HOMO → LUMO+2	19.5
				HOMO → LUMO+3	43.9
5	3.49	355.5	0.0893	HOMO-3 → LUMO	27.8
				HOMO-2 → LUMO	3.3
				HOMO-1 → LUMO	10.3
				HOMO → LUMO+1	16.6
				HOMO → LUMO+2	14.5
				HOMO → LUMO+3	22.3

Supplementary Figure 17 | Absorption spectroscopic features of PP dimer in Py-1-SWNT model with type 2 configuration. a, Oscillator strength vs. electronic transition energies with the experimental absorption spectrum of Py-1-SWNT. The transition numbers in **b** are shown in **a**. **b**, Excitation energies, oscillator strengths, and compositions of the transitions. The transitions whose oscillator strengths are less than 2% are not included.

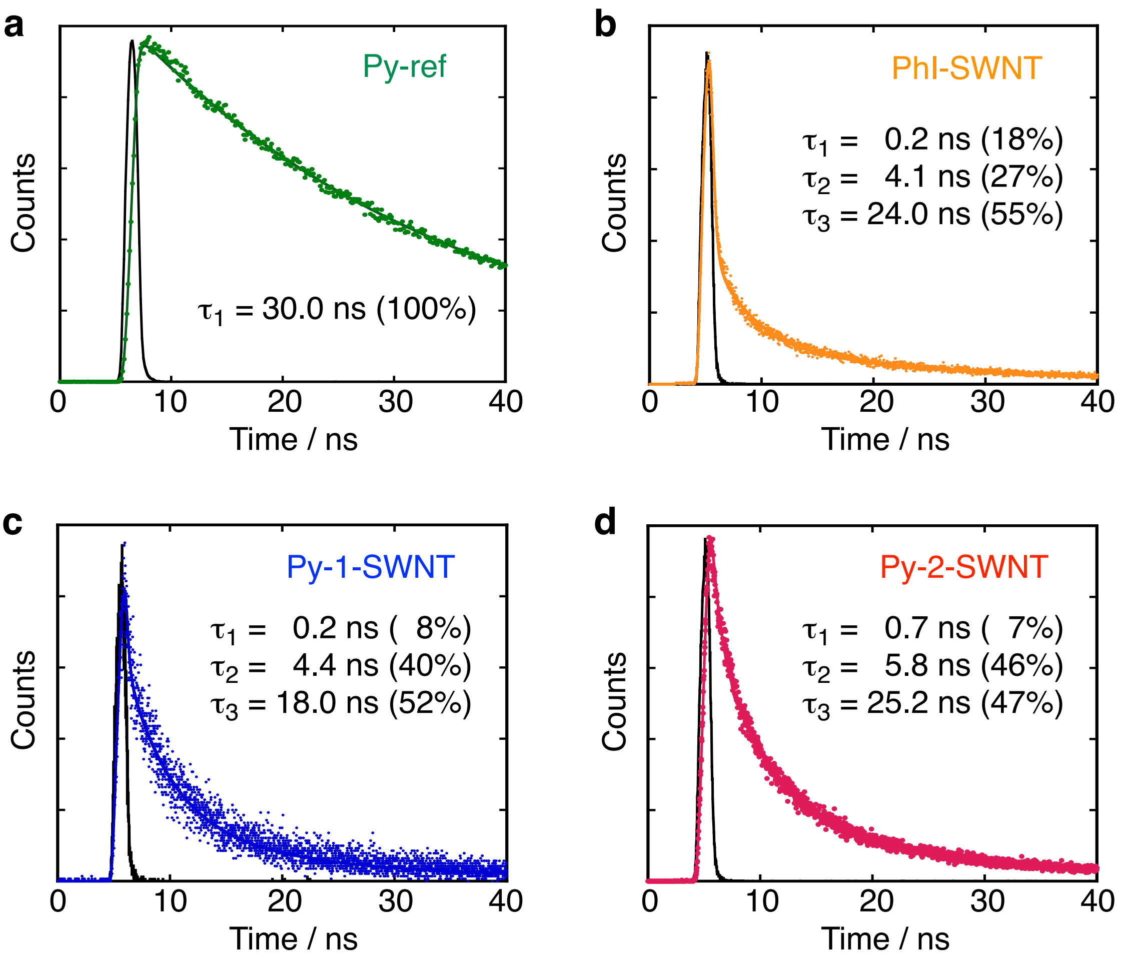

Supplementary Figure 18 | Fluorescence decays. **a**, Py-ref, **b**, PhI-SWNT, **c**, Py-1-SWNT and **d**, Py-2-SWNT measured in DMF. The excitation and monitoring wavelengths are 340 and 390 nm, respectively. Note that both the pyrene and SWNT are excited at 340 nm. The solid lines present decay fittings and the black lines show the instrumental response function. The fluorescence lifetimes with the ratios are given in the figures. Because the fluorescence decay profiles of Py-1-SWNT and Py-2-SWNT are similar to that of PhI-SWNT, all three components in the fluorescence decays of Py-1-SWNT and Py-2-SWNT can be reasonably attributed to the excited SWNTs that are known to show emissions due to the trapping of excitation energy by defect sites in the nanotube structure.[4]

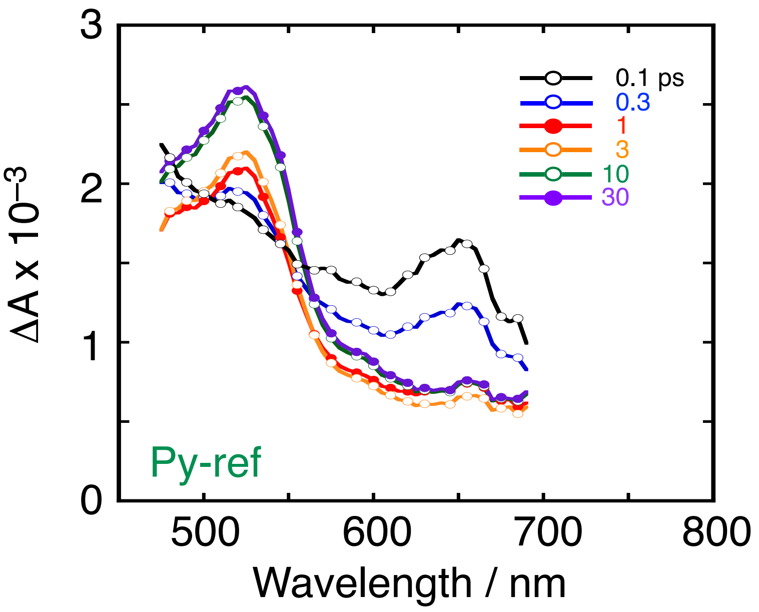

Supplementary Figure 19 I Femtosecond TA spectra of Py-ref in DMF. Several time delays between 0.1 and 30 ps are listed. Excitation wavelength is 350 nm. The spectra showed an instantaneously formed (< 0.1 ps) transient species with a maximum around 650 nm. This state is metastable and formation of another species follows, which is characterized by a positive signal at 530 nm and a long lifetime (> 6 ns, i.e., beyond the time limit of the present system for the TA measurements). We can assign these two species to the second and first singlet excited states, respectively.[5] The long decay lifetime of the second species (> 6 ns) is consistent with the emission lifetime of Py-ref (30 ns, Supplementary Fig. 18).

Supplementary References

(1) Stephenson, J. J., Hudson, J. L., Azad, S. & Tour, J. M. Individualized single walled carbon nanotubes from bulk material using 96% sulfuric acid as solvent. *Chem. Mater.* **18**, 374–377 (2005).

(2) Lolli, G., *et al.* Tailoring (*n*,*m*) structure of single-walled carbon nanotubes by modifying reaction conditions and the nature of the support of CoMo catalysts. *J. Phys. Chem. B* **110**, 2108–2115 (2006).

(3) Liu, H., Tanaka, T., Urabe, Y. & Kataura, H. High-efficiency single-chirality separation of carbon nanotubes using temperature-controlled gel chromatography. *Nano Lett.* **13**, 1996–2003 (2013).

(4) Lin, Y., *et al.* Visible luminescence of carbon nanotubes and dependence on functionalization. *J. Phys. Chem. B* **109**, 14779–14782 (2005).

(5) Gritsan, N. P., Pritchina, E. A., Barabanov, I. I., Burdzinski, G. T. & Platz, M. S. Excited-state dynamics in the covalently linked systems: pyrene-$(CH_2)_n$-aryl azide. *J. Phys. Chem. C* **113**, 11579–11589 (2009).
